# Synthetic Active Site Model of the [NiFeSe] Hydrogenase

**DOI:** 10.1002/chem.201500311

**Published:** 2015-04-02

**Authors:** Claire Wombwell, Erwin Reisner

**Affiliations:** [a] Christian Doppler Laboratory for Sustainable SynGas Chemistry, Department of Chemistry, University of Cambridge Lensfield Road, Cambridge CB2 1EW (UK) E-mail: reisner@ch.cam.ac.uk Homepage: http://www-reisner.ch.cam.ac.uk/

**Keywords:** active sites, enzyme models, hydrogenase, selenium, structural models

## Abstract

A dinuclear synthetic model of the [NiFeSe] hydrogenase active site and a structural, spectroscopic and electrochemical analysis of this complex is reported. [NiFe(‘S_2_Se_2_’)(CO)_3_] (H_2_‘S_2_Se_2_’=1,2-bis(2-thiabutyl-3,3-dimethyl-4-selenol)benzene) has been synthesized by reacting the nickel selenolate complex [Ni(‘S_2_Se_2_’)] with [Fe(CO)_3_bda] (bda=benzylideneacetone). X-ray crystal structure analysis confirms that [NiFe(‘S_2_Se_2_’)(CO)_3_] mimics the key structural features of the enzyme active site, including a doubly bridged heterobimetallic nickel and iron center with a selenolate terminally coordinated to the nickel center. Comparison of [NiFe(‘S_2_Se_2_’)(CO)_3_] with the previously reported thiolate analogue [NiFe(‘S_4_’)(CO)_3_] (H_2_‘S_4_’=H_2_xbsms=1,2-bis(4-mercapto-3,3-dimethyl-2-thiabutyl)benzene) showed that the selenolate groups in [NiFe(‘S_2_Se_2_’)(CO)_3_] give lower carbonyl stretching frequencies in the IR spectrum. Electrochemical studies of [NiFe(‘S_2_Se_2_’)(CO)_3_] and [NiFe(‘S_4_’)(CO)_3_] demonstrated that both complexes do not operate as homogenous H_2_ evolution catalysts, but are precursors to a solid deposit on an electrode surface for H_2_ evolution catalysis in organic and aqueous solution.

## Introduction

**Figure 1:**
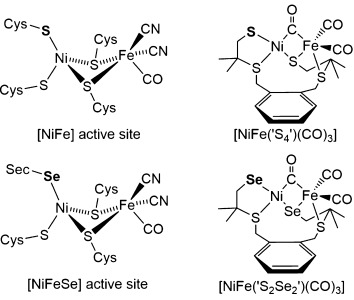
Representation of the active-site structures of a [NiFe] and [NiFeSe] hydrogenase in a reduced, active (Ni-SI) state and the corresponding synthetic models [NiFe(‘S_4_’)(CO_3_)][15d] and [NiFe(‘S_2_Se_2_’)(CO_3_)] (this work).

The depletion of fossil fuel reserves, the increasing levels of atmospheric CO_2_, and the need for energy security drive the development of new approaches to produce a renewable energy vector such as H_2_.[1] Inexpensive, stable, and efficient H_2_ generation catalysts are needed to produce sustainable H_2_ from water in the long term.[2] Hydrogenases are reversible H_2_ production catalysts and display remarkably high turnover frequencies of over 10^3^ s^−1^ at a small overpotential.[3] This incredible activity is achieved using the abundant metals nickel and iron in the hydrogenase active site.[4] [NiFeSe] hydrogenases are a subclass of the [NiFe] hydrogenases, where a selenocysteine (Sec) residue is terminally coordinated to the nickel center instead of a cysteine (Cys) in the enzyme active site (Figure 1).[5] [NiFeSe] hydrogenases have emerged as particularly suitable catalysts for H_2_ evolution,[6] because they exhibit high catalytic activities for H_2_ generation in the presence of H_2_ and fast reactivation from O_2_ inactivation when compared with other hydrogenases.[7] These advantageous properties make [NiFeSe] hydrogenases attractive for use in H_2_O splitting systems, and have allowed for their exploitation in a number of efficient photocatalytic H_2_ production schemes.[7e, 8]

As with other selenium containing enzymes,[9] it is still unclear what role selenium plays in the [NiFeSe] hydrogenases.[6] The Sec residue may affect the electronic and steric properties of the bimetallic core at the active site. Crystallographic evidence suggests that the Sec residue in the [NiFeSe] hydrogenase behaves as a proton relay during catalytic H_2_ cycling, carrying protons to and from the active site.[5] It is the Cys residue in the same position in the [NiFe] hydrogenase that was proposed to be the proton relay (Figure 1).[10] The unique reactivity of the [NiFeSe] hydrogenase with O_2_ may be the reason for its fast reactivation from O_2_ inactivation. When a conventional [NiFe] hydrogenase reacts with O_2_, the nickel center is oxidized to nickel(III) and an oxygen containing ligand takes the bridging position between the nickel and the iron centers.[11] In the O_2_ oxidized [NiFeSe] hydrogenase, however, the nickel center is not oxidized and no bridging ligand is observed between the two metal centers.[7g, 12] Crystallographic evidence suggests that it is the Sec selenium and, in some cases, Cys sulfur that is oxidized in [NiFeSe] hydrogenases.[12c,d, 13]

It has been well-established that the protein structure surrounding an active site affects the reactivity of an enzyme,[14] and biomimetic molecules can be employed to learn about the structural and functional properties of the active site.[15] Our aim is to explore the effect of selenium on the enzyme active site using small molecule model chemistry.

A large number of dinuclear models of the active site of the conventional [NiFe] hydrogenases have previously been reported following the determination of the X-ray crystal structure of the enzyme.[1c, 15d,f,h,m,n, 16] Several structural and functional [NiFe] hydrogenase models have been prepared using the nickel precursor complex [Ni(‘S_4_’)] (H_2_‘S_4_’=H_2_xbsms=1,2-bis(4-mercapto-3,3-dimethyl-2-thiabutyl)benzene).[15d,f, 16b, 17] [Ni(‘S_4_’)] was initially used as precursor to assemble a number of thiolate bridged [NiFe] complexes[16b] and dinuclear [NiRu] complexes.[17b–d,f] The [NiRu] complexes were some of the first functional hydrogenase models reported to catalyze H_2_ production.[17b–d,f] Since then, two structural and functional [NiFe] hydrogenase models that use [Ni(‘S_4_’)] as a precursor have been reported.[15d,f] One of these, the asymmetrical [NiFe] complex [NiFe(‘S_4_’)(CO)_3_], is the only dinuclear [NiFe] hydrogenase model that contains a thiolate donor terminally bound to the nickel center (Figure 1).[15d]

We have recently reported on a series of nickel complexes as structural models of the nickel center in the active sites of the [NiFeSe] hydrogenases.[18] Herein, we report a dinuclear [NiFeSe] hydrogenase active site model, which includes an iron carbonyl to replicate the core features of the enzyme active site. The synthesis, characterization, and activity of [NiFe(‘S_2_Se_2_’)(CO)_3_] is reported and it has been compared with the previously reported[15d] thiolate analogue [NiFe(‘S_4_’)(CO)_3_] to determine the influence of the chalcogenate donor on the properties of the complex (Figure 1). A detailed structural, spectroscopic, and electrochemical analysis as well as a comparison with the hydrogenase active site is presented.

## Results and Discussion

### Synthesis and characterization of [NiFe(‘S_2_Se_2_’)(CO)_3_]

**Figure 2:**
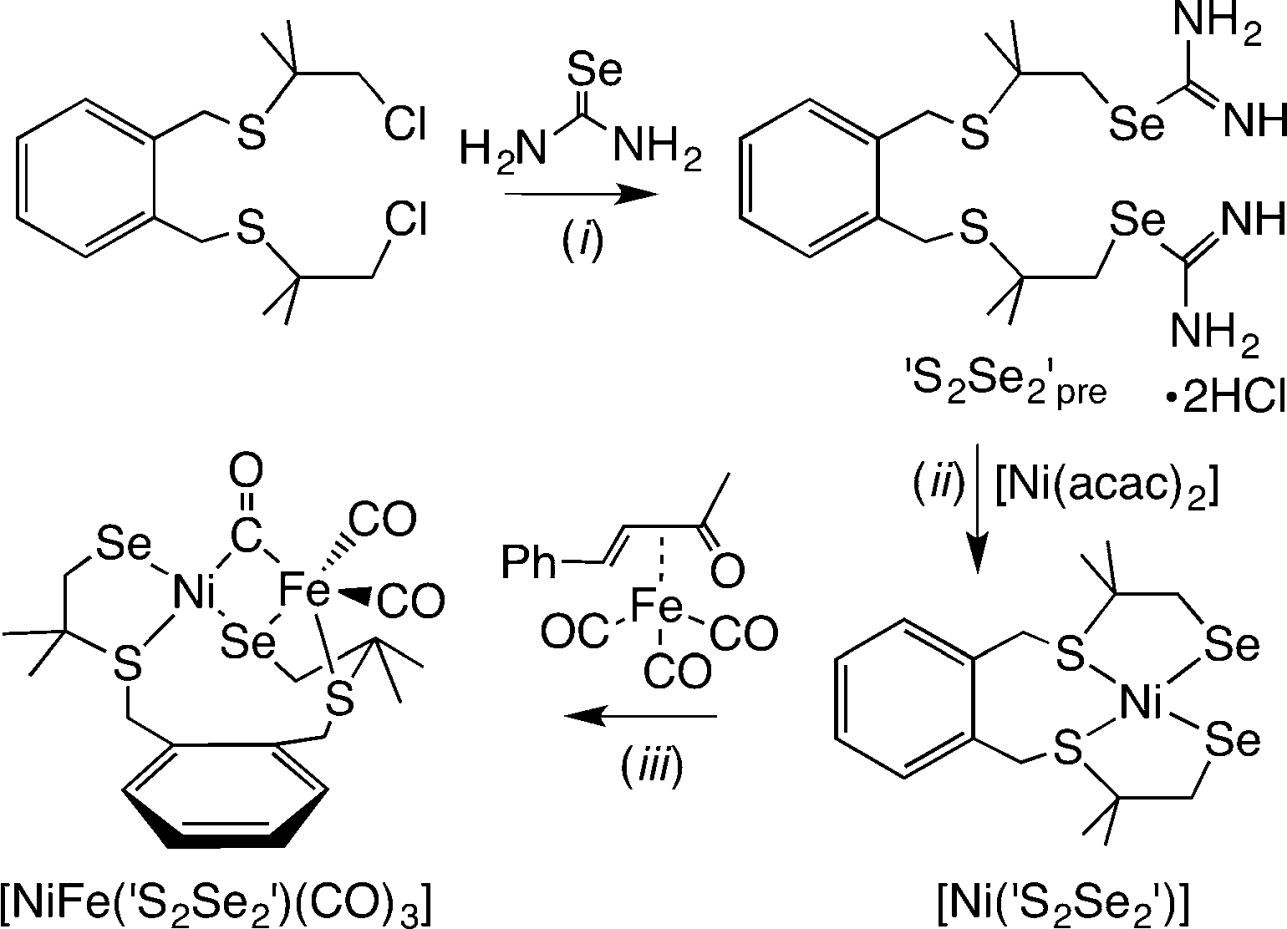
Synthesis of the ‘S_2_Se_2_’ ligand precursor ‘S_2_Se_2_’_pre_, the mononuclear Ni complex [Ni(‘S_2_Se_2_’)] and the [NiFeSe] hydrogenase active site mimic [NiFe(‘S_2_Se_2_’)(CO)_3_]. Conditions: *i*) ethanol, RT, 61 % yield; *ii*) 2 equiv NMe_4_OH⋅5 H_2_O, ethanol, reflux, 83 % yield; *iii*) dichloromethane, RT, 43 % yield.

[NiFe(‘S_2_Se_2_’)(CO)_3_] was synthesized following the procedure shown in Figure 2. Bis(3-chloro-2,2-methyl-1-thiapropyl)-*o*-xylene[17a] was reacted with two equivalents of selenourea in ethanol at room temperature to give the selenouronium compound ‘S_2_Se_2_’_pre_. This ligand precursor was isolated as a white powder in 61 % yield and characterized by ^1^H and ^13^C NMR spectroscopy, mass spectrometry, and elemental analysis (Supporting Information, Figures S1–S5). [Ni(‘S_2_Se_2_’)] was prepared by refluxing [Ni(acac)_2_] (acac=acetylacetonato) with one equivalent of ‘S_2_Se_2_’_pre_ and two equivalents of NMe_4_OH⋅5 H_2_O as a base under inert conditions for 1 h in ethanol. The solvent volume was decreased to precipitate the product as a green solid, which was separated by filtration and recrystallized by slow diffusion of hexane into a dichloromethane solution of the complex to give [Ni(‘S_2_Se_2_’)] in 83 % yield. The green complex was characterized by ^1^H and ^13^C NMR, IR, and electronic absorption spectroscopy, mass spectrometry, elemental analysis, and single-crystal X-ray diffraction (Supporting Information, Figures S6–S10). Reaction of [Ni(‘S_2_Se_2_’)] with one equivalent of [Fe(CO)_3_(bda)] (bda=benzylideneacetone) in dichloromethane readily formed the red solid [NiFe(‘S_2_Se_2_’)(CO)_3_] at room temperature, which was isolated in 43 % yield. [NiFe(‘S_2_Se_2_’)(CO)_3_] was characterized using ^1^H and ^13^C NMR, IR, and electronic absorption spectroscopy, mass spectrometry, elemental analysis, and single-crystal X-ray diffraction (Supporting Information, Figures S11–S16).

### Structural characterization

**Figure 3:**
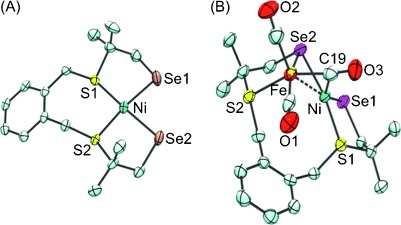
Single-crystal X-ray structures of A) [Ni(‘S_2_Se_2_’)] (*R*1 = 3.11 %) and B) [NiFe(‘S_2_Se_2_’)(CO)_3_] (*R*1 = 2.98 %). Ellipsoids are set at 50 % probability; hydrogen atoms have been omitted for clarity. The atom labeling shows all atoms except for carbon. One of two crystallographically independent molecules is shown for [Ni(‘S_2_Se_2_’)]. A disordered solvent pentane molecule is omitted in the structure of [NiFe(‘S_2_Se_2_’)(CO)_3_].

Single crystals of [Ni(‘S_2_Se_2_’)] were grown by liquid diffusion of hexane into a saturated dichloromethane solution of the complex and the X-ray crystal structure is shown in Figure 3 A. Complex [Ni(‘S_2_Se_2_’)] crystallizes in the space group *P*2_1_/*n* with two crystallographically independent molecules per asymmetric unit. Selected distances and angles for [Ni(‘S_2_Se_2_’)] and the previously reported [Ni(‘S_4_’)][17a] are summarized in Table 1. Crystal data and refinement details for [Ni(‘S_2_Se_2_’)] are given in the Supporting Information, Table S1. [Ni(‘S_2_Se_2_’)] contains nickel(II) coordinated to two selenolate and two thioether donors with square-planar geometry around the nickel center. The bond distances are as expected: the nickel selenolate distances of 2.295(8) Å in [Ni(‘S_2_Se_2_’)] are longer than the nickel thiolate distances of 2.184(3) Å in [Ni(‘S_4_’)],[17a] as observed with other nickel thiolate/selenolate complexes.[18] The five-membered rings in [Ni(‘S_2_Se_2_’)] constrain the Se-Ni-S angles negligibly to 89.3(4)° and the seven-membered ring of the xylenediyl group pushes the S1-Ni-S2 angle to 99.4(2)°_._ There is a small tetrahedral distortion from the square plane around the nickel center with an angle of 11.1±1° between the planes Se1-Ni-S1 and Se2-Ni-S2. The corresponding tetrahedral distortion in [Ni(‘S_4_’)] is smaller at 4.31±4°.[17a]

**Table 1:** Selected bond distances and angles for [Ni(‘S_2_Se_2_’)] and [Ni(‘S_4_’)][17a] model complexes^[a]^

[Ni(‘S_2_Se_2_’)]	Distance [Å]	[Ni(‘S_4_’)]	Distance [Å]
Ni–Se1	2.3023(7)	Ni–S′1	2.1802(6)
Ni–Se2	2.2872(7)	Ni–S′2	2.1869(6)
Ni–S1	2.192(1)	Ni–S1	2.1909(6)
Ni–S2	2.184(1)	Ni–S2	2.1756(5)

[Ni(‘S_2_Se_2_’)]	Angle [°]	[Ni(‘S_4_’)]	Angle [°]
Se1-Ni-Se2	84.21(2)	S′1-Ni-S′2	84.05(2)
Se2-Ni-S2	89.69(3)	S′2-Ni-S2	87.11(2)
S1-Ni-S2	97.16(4)	S1-Ni-S2	100.75(2)
Se1-Ni-S1	88.93(3)	S′1-Ni-S1	87.92(2)
S1-Ni-Se2	173.12(4)	S1-Ni-S′2	171.45(2)
Se1-Ni-S2	167.31(4)	S′1-Ni-S′2	170.72(2)

[a] Distances and angles for one of two molecules in the asymmetric unit in both cases; S=thioether, S′=thiolate donor.

Single crystals of [NiFe(‘S_2_Se_2_’)(CO)_3_] were grown from liquid diffusion of pentane into a saturated dichloromethane solution of the complex. The complex crystallizes in the space group *P*2_1_/*n* with a disordered pentane solvent molecule in half occupancy (Experimental Section and Supporting Information, Table S1). The X-ray crystal structure of [NiFe(‘S_2_Se_2_’)(CO)_3_] is shown in Figure 3 B and it is structurally similar to the previously reported [NiFe(‘S_4_’)(CO)_3_].[15d] Selected bond distances and angles for [NiFe(‘S_2_Se_2_’)(CO)_3_] and [NiFe(‘S_4_’)(CO)_3_][15d] are shown in Table 2. One of the ‘S_2_Se_2_’ selenolate donor ligands bridges the nickel and the iron centers, whereas the other remains terminally coordinated to the nickel center. One of the three carbonyl ligands takes up a bridging position between the nickel and the iron centers. One of the ‘S_2_Se_2_’ thioether donors has become uncoordinated from the nickel center and coordinates instead to iron whilst the other remains coordinated to nickel. The coordination geometry around the nickel center is distorted tetrahedral and around the iron center is square-based pyramidal.

**Table 2:** Selected bond distances and angles for [NiFe(‘S_2_Se_2_’)(CO)_3_] and [NiFe(‘S_4_’)(CO)_3_][15d] model complexes^[a]^

[NiFe(‘S_2_Se_2_’)(CO)_3_]	Distances [Å]	[NiFe(‘S_4_’)(CO)_3_]	Distances [Å]
Ni–Fe	2.4480(4)	Ni–Fe	2.4262(2)
Ni–Se1	2.2802(4)	Ni–S′1	2.1644(3)
Ni–S1	2.1940(6)	Ni–S1	2.1942(3)
Ni–Se2	2.2947(3)	Ni–S′2	2.1749(3)
Ni–C19	2.033(3)	Ni–C19	2.035(1)
Fe–Se2	2.3699(4)	Fe–S′2	2.2567(3)
Fe–C19	1.822(3)	Fe–C19	1.823 (1)
Fe–S2	2.3088(7)	Fe–S2	2.3058(4)
C19–O3	1.168(3)	C19–O3	1.165 (1)

[NiFe(‘S_2_Se_2_’)(CO)_3_]	Angle [°]	[NiFe(‘S_4_’)(CO)_3_]	Angle [°]
Se1-Ni-S1	94.28 (2)	S′1-Ni-S1	93.38(4)
Se1-Ni-Se2	97.37 (1)	S′1-Ni-S′2	104.60(4)
Se1-Ni-C19	142.77(8)	S′1-Ni-C19	135.5(1)
Se1-Ni-Fe	156.40(1)	S′1-Ni-Fe	162.50(4)
C19-Fe-Ni	54.51(8)	C19-Fe-Ni	67.5(1)
C19-Ni-Fe	46.85(8)	C19-Ni-Fe	43.3(1)
Ni-Se2-Fe	63.29 (1)	Ni-S′2-Fe	67.25(3)

[a] S=thioether, S′=thiolate donor.

[NiFe(‘S_2_Se_2_’)(CO)_3_] possesses a number of the key structural features of the [NiFeSe] hydrogenase active site including one nickel and one iron center held together by two bridging ligands and, most importantly, a selenolate donor terminally coordinated to the nickel center. All metal–selenium bonds in [NiFe(‘S_2_Se_2_’)(CO)_3_] are approximately 0.1 Å longer than the equivalent metal–sulfur bonds in [NiFe(‘S_4_’)(CO)_3_].[15d]

The distance between the nickel and the terminal selenium donor in [NiFe(‘S_2_Se_2_’)(CO)_3_] is 2.28 Å and the nickel selenolate bond distance was reported as 2.46 Å in the [NiFeSe] hydrogenase from *Desulfomicrobium baculatum* at 2.15 Å resolution.[5] The Ni–Se distance in the structure of the active *Desulfomicrobium baculatum* [NiFeSe] hydrogenase is 0.25 Å longer than the equivalent Ni–S distance in the active *Desulfovibrio vulgaris* Miyazaki F [NiFe] hydrogenase structure, although a significant error can be expected in the enzyme structure owing to a high temperature factor for the Sec residue.[5, 19] Theoretical modeling studies show the Ni–Se distance to be 0.11 Å longer than the equivalent Ni–S distance in the [NiFe] hydrogenase, which correlates well with the bond distances observed in the small molecule models studied herein.[20] The introduction of selenium does not significantly affect any of the other distances in the model complexes. The Ni–Fe distance is only marginally longer (0.02 Å) in [NiFe(‘S_2_Se_2_’)(CO)_3_] than in [NiFe(‘S_4_’)(CO)_3_] and the distances between the metal, and the bridging carbonyl carbon are almost identical in both complexes. Theoretical modeling also showed that the introduction of selenium into the hydrogenase active site would not affect any other bond distances.[20] The bond angles in the structure of [NiFe(‘S_2_Se_2_’)(CO)_3_] are similar to those in [NiFe(‘S_4_’)(CO)_3_]. The angle between the planes of Ni-C19-Fe (Figure 3 B) and Ni-Se2-Fe is 71.1(1)° and the equivalent angle in [NiFe(‘S_4_’)(CO)_3_] is 69.7°.[15d]

The diamagnetism of [NiFe(‘S_2_Se_2_’)(CO)_3_] as well as the short nickel–iron distance indicate that there is a bond between the two metal centers, which is supported by previously reported density functional theory studies of [NiFe(‘S_4_’)(CO)_3_].[15d]

### Spectroscopic characterization

**Figure 4:**
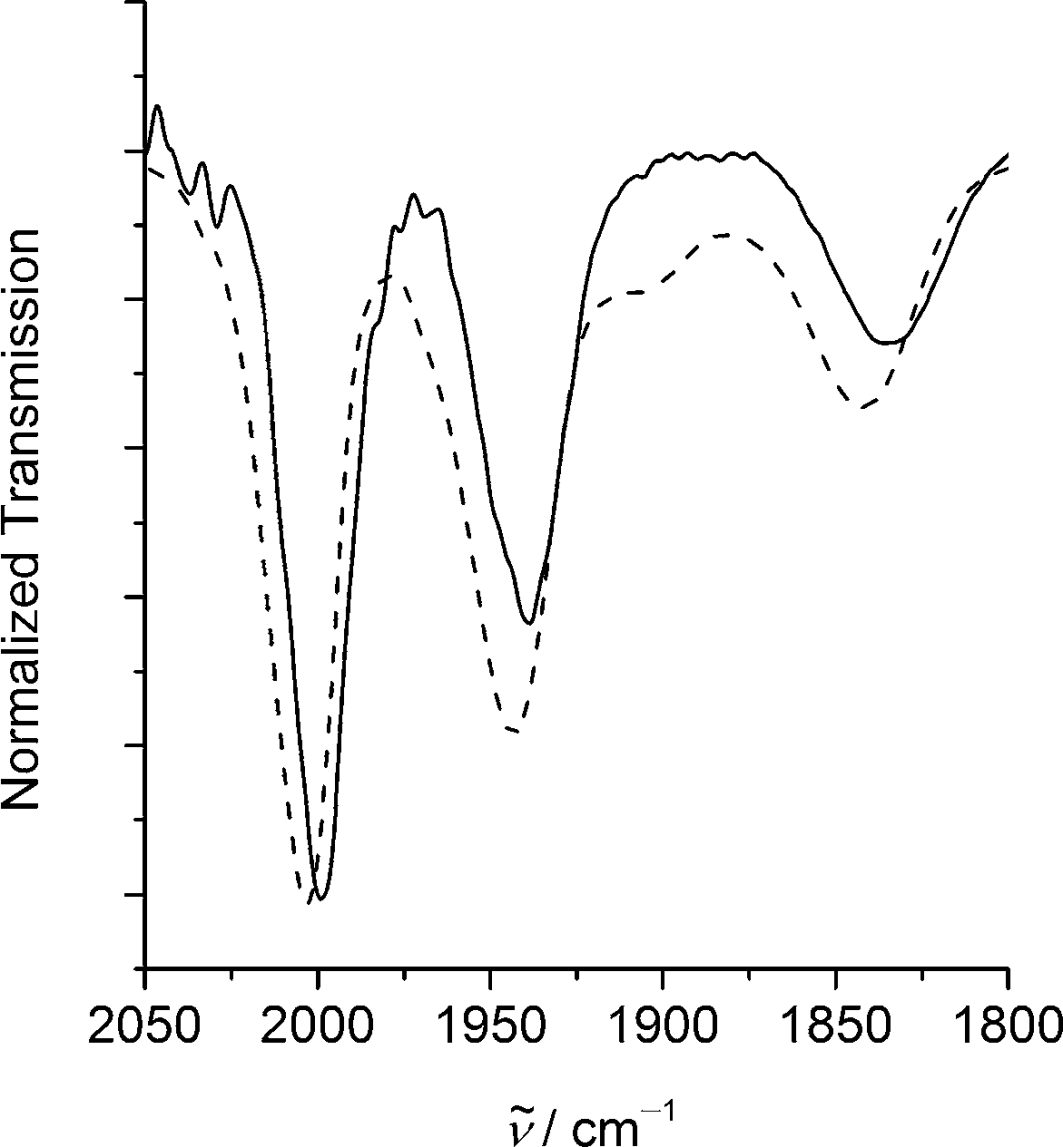
Attenuated total reflectance FTIR spectra of [NiFe(‘S_2_Se_2_’)(CO)_3_] (—) and [NiFe(‘S_4_’)(CO)_3_] (- - - -).

Figure 4 shows the carbonyl region of the IR spectrum of [NiFe(‘S_2_Se_2_’)(CO)_3_] and [NiFe(‘S_4_’)(CO)_3_]. Complex [NiFe(‘S_2_Se_2_’)(CO)_3_] displays three carbonyl stretching bands: two at 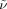 = 2000 and 1942 cm^−1^ attributable to the terminally coordinated carbonyls and one at 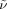 = 1835 cm^−1^ for the bridging carbonyl. All three CO stretching frequencies for [NiFe(‘S_2_Se_2_’)(CO)_3_] are 1–7 cm^−1^ lower than those for [NiFe(‘S_4_’)(CO)_3_] (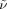 = 2003, 1943, and 1842 cm^−1^),[15d] indicating increased π back-donation from the metal d orbitals to the carbonyl π* antibonding orbital, owing to increased electron density at the metal centers from selenium. The same trend is observed in the [NiFeSe] hydrogenase active sites. The stretching frequencies of the CO ligand terminally coordinated to iron in the Ni–C states of the [NiFeSe] hydrogenase from *Desulfovibrio vulgaris* Miyazaki F (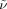 = 1948 and 1925 cm^−1^_,_ two different isoforms)[7g] and *Desulfovibrio vulgaris* Hildenborough (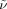 = 1915 and 1900 cm^−1^, two different isoforms)[7f] are lower than those observed for the [NiFe] hydrogenases (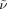 = 1961–1949 cm^−1^).[21] Furthermore, a CO inhibited *Desulfovibrio vulgaris* Hildenborough [NiFeSe] hydrogenase, in which there is a CO ligand coordinated to the active site nickel center, exhibits a 5–15 cm^−1^ shift to lower frequencies (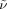 = 2052, 2042 cm^−1^, two different isoforms) compared to CO inhibited conventional [NiFe] hydrogenases from *Desulfovibrio fructosovorans*, *Desulfovibrio vulgaris* Miyazaki F, and *Chromatium vinosum*.[7f, 11e, 21b, 22]

**Figure 5:**
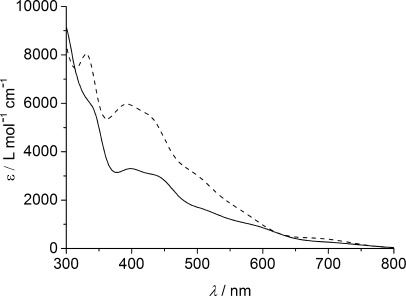
Electronic absorption spectra of [NiFe(‘S_2_Se_2_’)(CO)_3_] (—) and [NiFe(‘S_4_’)(CO)_3_] (- - - -) in DMF.

The electronic absorption spectra of [NiFe(‘S_2_Se_2_’)(CO)_3_] and [NiFe(‘S_4_’)(CO)_3_] in DMF are shown in Figure 5, and the corresponding data are given in the Supporting Information, Table S2. There are several strong bands in the visible region of the spectrum of [NiFe(‘S_2_Se_2_’)(CO)_3_]. The absorption bands exhibited by [NiFe(‘S_2_Se_2_’)(CO)_3_] are all red-shifted relative to [NiFe(‘S_4_’)(CO)_3_], as expected due to the increased size and polarizability of selenium relative to sulfur.

### Electrochemical characterization

**Figure 6:**
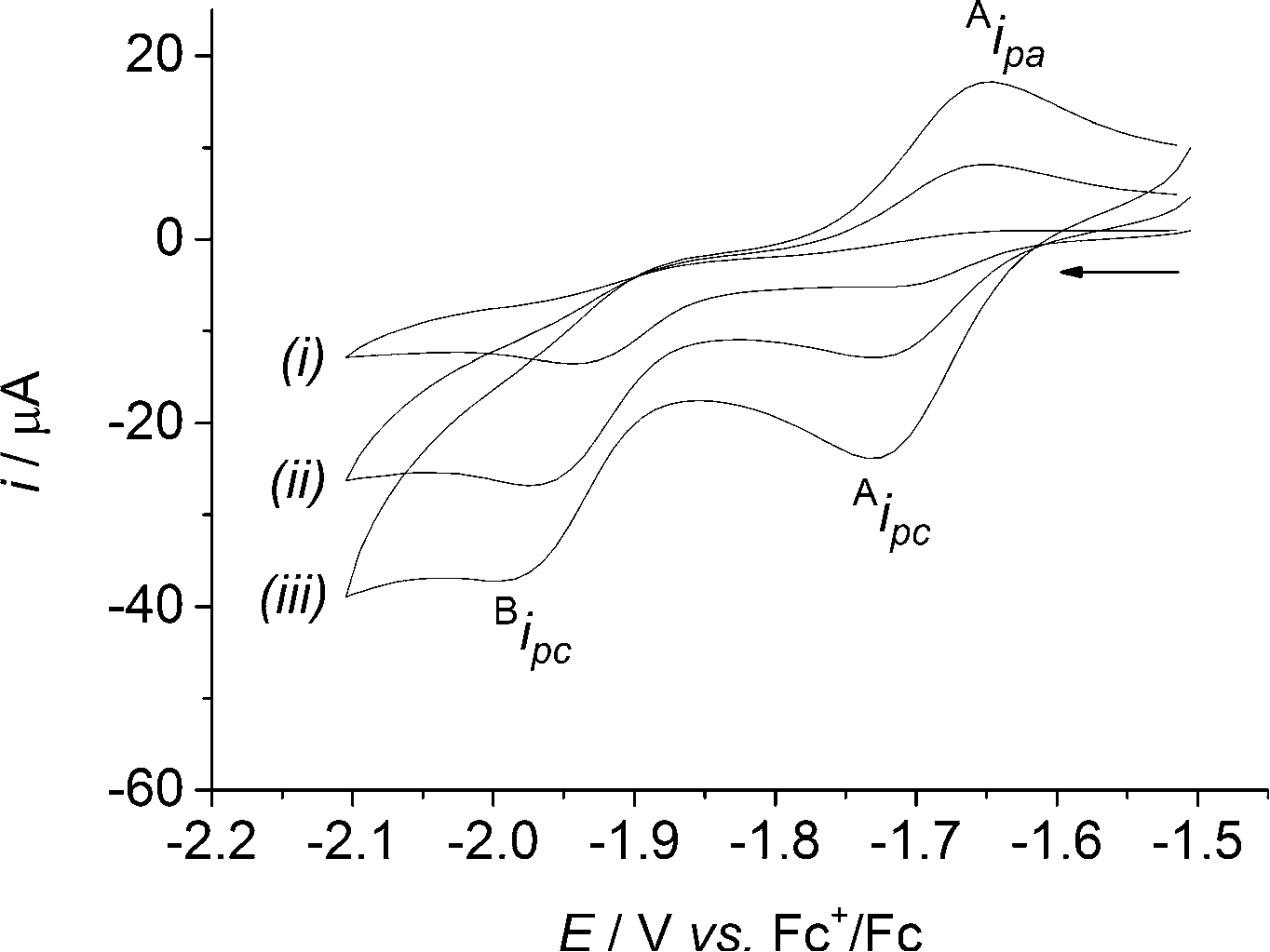
Cyclic voltammograms of [NiFe(‘S_2_Se_2_’)(CO)_3_] (1 mm) in acetonitrile (0.1 m *n*-Bu_4_NBF_4_) at a scan rate of *i*) 100, *ii*) 500, and *iii*) 1000 mV s^−1^.

The cyclic voltammograms of [NiFe(‘S_2_Se_2_’)(CO)_3_] and [NiFe(‘S_4_’)(CO)_3_] (1 mm) exhibit two irreversible reduction waves in an acetonitrile electrolyte solution at 100 mV s^−1^ (Figure 6; Supporting Information, Figure S17). The first reduction wave, wave A, at *E*_pc_=−1.72 and −1.76 V is followed by a second wave, wave B, at −1.98 V and −1.99 V vs. Fc^+^/Fc for [NiFe(‘S_2_Se_2_’)(CO)_3_] and [NiFe(‘S_4_’)(CO)_3_], respectively. The first reduction wave becomes almost reversible in both complexes at higher scan rates, with a peak current ratio (^A^*i*_pa_/^A^*i*_pc_) of approximately 0.8 at 1000 mV s^−1^. In DMF electrolyte solution and at a scan rate of 100 mV s^−1^, the two reduction waves are observed at *E*_pc_=−1.72 and −1.98 V for [NiFe(‘S_2_Se_2_’)(CO)_3_] and at *E*_pc_=−1.79 and −2.01 V vs. Fc^+^/Fc for [NiFe(‘S_4_’)(CO)_3_]. Both the first and second reduction waves of [NiFe(‘S_2_Se_2_’)(CO)_3_] are shifted to more anodic potentials compared to [NiFe(‘S_4_’)(CO)_3_], which is consistent with the substitution of a thiolate by a selenolate donor ligand in a number of other transition-metal complexes.[15e,g,i–l, 23] For example, this trend was observed with [FeFe] hydrogenase model complexes with selenolate containing bridging ligands compared to the same complex with thiolate containing bridging ligands.[15e,g,i–l]

The same trend is also exhibited by complexes [Ni(‘S_2_Se_2_’)] and [Ni(‘S_4_’)], which exhibit one irreversible reduction wave in DMF at *E*_pc_=−1.87 V and −1.99 V vs. Fc^+^/Fc at 100 mV s^−1^, respectively (Supporting Information, Figure S18). This reduction wave was previously assigned for [Ni(‘S_4_’)] as the reduction of the nickel center.[17c]

[NiFe(‘S_4_’)(CO)_3_] was previously reported to behave as an electrocatalyst for H_2_ production in an acetonitrile electrolyte solution with trifluoroacetic acid (TFA).[15d] We have repeated these experiments and also observed a catalytic wave in the cyclic voltammogram of [NiFe(‘S_4_’)(CO)_3_] in an acetonitrile electrolyte solution in the presence of TFA using a glassy carbon working electrode. The peak current of this catalytic wave increases with increasing concentrations of the acid. However, a control experiment showed that a comparable catalytic response was also observed when cyclic voltammograms were recorded on a bare glassy carbon electrode under the same conditions but in the absence of [NiFe(‘S_4_’)(CO)_3_]. The linear sweep voltammograms on a glassy carbon electrode in the presence and absence of [NiFe(‘S_4_’)(CO)_3_] with increasing concentrations of TFA in acetonitrile are shown in the Supporting Information, Figure S19. Thus, [NiFe(‘S_4_’)(CO)_3_] does not display significant catalytic activity under these conditions (see further discussion below).

The catalytic activity of the complexes was thus assessed using DMF as a solvent as the pKa of many organic acids in DMF is significantly higher than in acetonitrile.[24] No catalytic current enhancement was detected using [NiFe(‘S_2_Se_2_’)(CO)_3_] or [NiFe(‘S_4_’)(CO)_3_] in DMF in the presence of acetic acid or benzoic acid, but with the stronger TFA, H_2_ production activity was observed (Supporting Information, Figure S20). A catalytic wave appeared, which showed an increase in current with increasing acid concentrations, whereas voltammograms on a bare glassy carbon electrode under the same conditions with no complex gave negligible current enhancement (Supporting Information, Figure S21).

**Figure 7:**
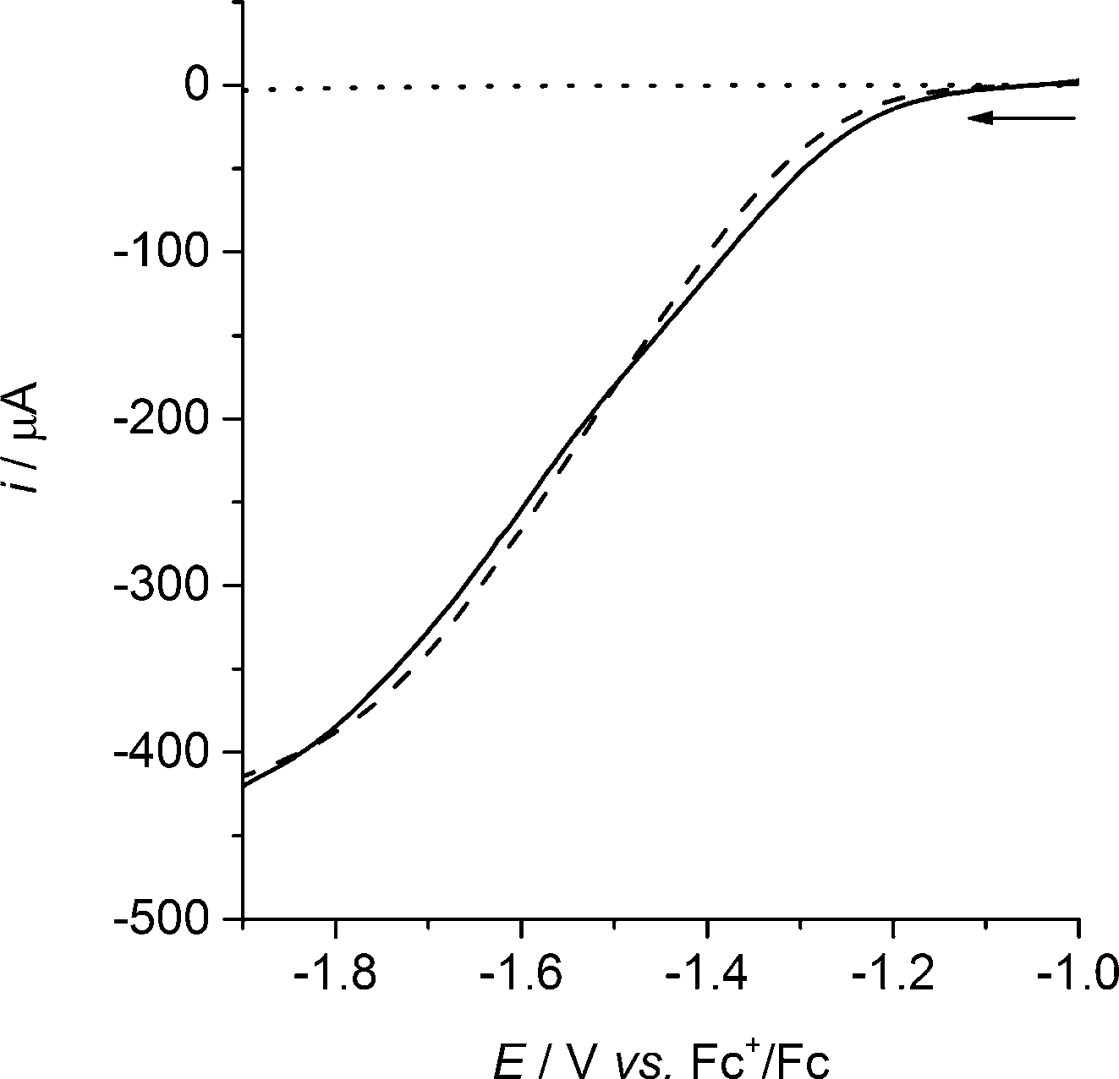
Linear sweep voltammograms of the solid deposit formed from electrodeposition of [NiFe(‘S_2_Se_2_’)(CO)_3_] in the presence (—) and absence (- - - -) of dissolved [NiFe(‘S_2_Se_2_’)(CO)_3_] (1 mm) on a glassy carbon working electrode in DMF (0.1 m *n*-Bu_4_NBF_4_) containing TFA (100 mm) at a scan rate of 100 mV s^−1^. The response of an unmodified (bare) glassy carbon working electrode in TFA/DMF (••••) is also shown (recorded in the absence of [NiFe(‘S_2_Se_2_’)(CO)_3_] in solution).

However, the catalytic response does not result from homogeneous catalysis, but a solid deposit on the electrode surface formed by the electrodeposition of either [NiFe(‘S_2_Se_2_’)(CO)_3_] or [NiFe(‘S_4_’)(CO)_3_]. Following cyclic voltammetry of the complex in DMF with increasing concentrations of TFA (up to 100 mm; Supporting Information, Figure S20) the working electrode was removed from the solution and rinsed with DMF. This electrode was then placed in a fresh electrolyte solution (rinse test) containing 100 mm of TFA without any NiFe complex in solution and the same catalytic response was observed as with the complex in solution (Figure 7; Supporting Information, Figure S22). The stability of the complexes in TFA/DMF solution in the absence of an applied potential was established using electronic absorption spectroscopy, confirming that a solid deposit is formed through electrodeposition (Supporting Information, Figure S23).

A comparable catalyst precursor activity was also observed for our previously reported mononuclear nickel thiolate/selenolate complexes.[18] Deposition of a growing number of first-row transition-metal complexes onto electrodes is being reported and the nature of the precursor complex affects the morphology and activity of the resulting heterogeneous catalyst.[2d, 18, 25]

**Figure 8:**
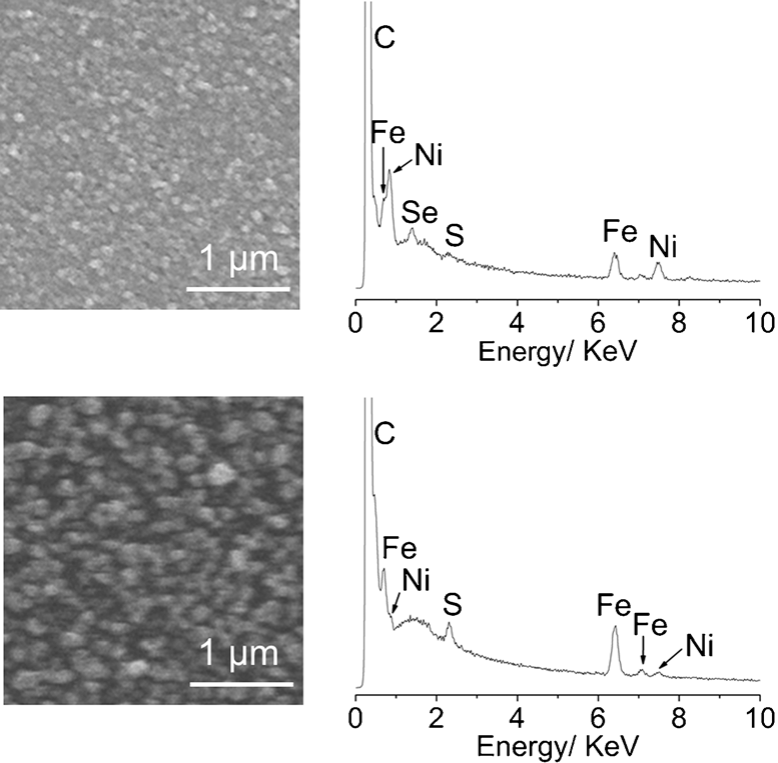
SEM and EDX analysis of films electrodeposited on a glassy carbon electrode from complexes [NiFe(‘S_2_Se_2_’)(CO)_3_] (top) and [NiFe(‘S_4_’)(CO)_3_] (bottom).

Thus, the composition of the deposit from [NiFe(‘S_2_Se_2_’)(CO)_3_] and [NiFe(‘S_4_’)(CO)_3_] was characterized to determine the nature of the catalytic species. A glassy carbon slide with a surface area of 1.6 cm^2^ was modified with the deposit through electrodeposition from a solution of [NiFe(‘S_2_Se_2_’)(CO)_3_] or [NiFe(‘S_4_’)(CO)_3_] (1 mm) in the presence of TFA (10 mm) in DMF at *E*_appl_ = −1.75 V vs. Fc^+^/Fc for 0.5 h. The modified electrode was then removed from the solution and rinsed with DMF (3 mL) before analysis. Scanning electron microscopy (SEM) analysis of the slides treated with either [NiFe(‘S_2_Se_2_’)(CO)_3_] or [NiFe(‘S_4_’)(CO)_3_] revealed that in both cases the electrode is entirely covered in a film of the deposit (Figure 8). Energy-dispersive X-ray spectroscopy (EDX) analysis confirmed that both films consist mainly of nickel and iron (Figure 8; Supporting Information, Table S3). There is sulfur (4 atom %) and selenium (16 atom %) in the film deposited from [NiFe(‘S_2_Se_2_’)(CO)_3_] and sulfur (13 atom %) in the film deposited from [NiFe(‘S_4_’)(CO)_3_]. Low levels of sulfur and selenium rule out the possibility that the bulk of the film material is a metal sulfide or metal selenide. Surface analysis by X-ray photoelectron spectroscopy (XPS) confirmed that the film surface is mostly comprised of nickel and iron, with small amounts of sulfur and/or selenium (Supporting Information, Table S3). The Ni 2p signals in the XPS spectra of both deposits at 874 and 856 eV with satellites at 880 and 862 eV correspond to Ni(OH)_2_ (Supporting Information, Figure S24). The surfaces were exposed to air before analysis so it is reasonable to assume that significant surface oxidation occurred. A small nickel(0) peak is visible in the Ni 2p spectrum of the deposit from [NiFe(‘S_2_Se_2_’)(CO)_3_] at 852 eV, which is possibly the catalytically active species. The Fe 2p signals in both deposits at 711 and 724 eV with satellites at 719 and 732 eV show that it is in the form of iron oxide and no resolvable iron(0) signal is observed.

The deposits from [NiFe(‘S_2_Se_2_’)(CO)_3_] and [NiFe(‘S_4_’)(CO)_3_] on a glassy carbon disk or fluorine-doped tin oxide (FTO) electrode (electrodeposited at −1.75 V vs. Fc^+^/Fc for 0.5 h in a 1 mm solution of the complex in DMF containing 10 mm TFA) were also shown to be electroactive for H_2_ evolution in an aqueous pH neutral phosphate solution (Supporting Information, Figure S25). The deposits show comparable activity to other nickel containing H_2_ production catalyst films formed from molecular precursors recently reported.[18, 25d] Controlled potential electrolysis of such films on an FTO electrode with a surface area of 1.6 cm^2^ confirmed the generation of H_2_ (headspace gas chromatography analysis).

## Conclusion

A synthetic structural model of the [NiFeSe] hydrogenase active site has been reported. The complex was synthesized using the nickel precursor complex [Ni(‘S_2_Se_2_’)], in which the nickel center is surrounded by two selenolate and two thioether donors. Complex [NiFe(‘S_2_Se_2_’)(CO)_3_] mimics several of the main structural features of the enzyme active site, including one nickel and one iron center held together by two bridging ligands and a selenolate donor terminally coordinated to the nickel center. Relevant distances and angles in [NiFe(‘S_2_Se_2_’)(CO)_3_] agree well with those found in the enzyme. The nickel–selenium distance is 0.1 Å longer and the nickel–iron distance in [NiFe(‘S_2_Se_2_’)(CO)_3_] only slightly longer than the analogous [NiFe] hydrogenase model complex [NiFe(‘S_4_’)(CO)_3_]. The metal–carbonyl bond lengths in the two complexes are almost identical. The differences in the spectroscopic properties of [NiFe(’S_2_Se_2_’)(CO)_3_] and [NiFe(‘S_4_’)(CO)_3_] illustrate the differences in their electronic structures. IR spectroscopy revealed that the carbonyl bands in [NiFe(‘S_2_Se_2_’)(CO)_3_] are all shifted to lower frequencies relative to [NiFe(‘S_4_’)(CO)_3_], indicating that the more electron-donating selenolate groups offer an increased electron density at the Fe center. The signals in the electronic absorption spectrum of [NiFe(‘S_2_Se_2_’)(CO)_3_] are shifted to lower energies than in [NiFe(‘S_4_’)(CO)_3_]. Extensive electrochemical studies revealed that both NiFe complexes do not behave as homogenous catalysts for H_2_ evolution, but are molecular precursors for active heterogeneous catalysts, which can be readily electrodeposited onto an electrode surface. Analysis of the solid deposits shows that these films contain nickel and iron with some sulfur and selenium. The deposit is electrocatalytically active for proton reduction in organic solvents with acid or aqueous pH neutral phosphate solution.

## Experimental Section

### Materials and methods

All of the complexes were synthesized using anhydrous anaerobic techniques using a Schlenk line unless otherwise noted. All starting materials were purchased from commercial suppliers in the highest available purity for all analytical measurements and used without further purification. Organic solvents were dried and deoxygenated prior to use. Bis(3-chloro-2,2-methyl-1-thiapropyl)-*o*-xylene and [Ni(‘S_4_’)],[17a] [Fe(CO)_3_bda],[26] and [NiFe(‘S_4_’)(CO)_3_][15d] have been synthesized using previously reported procedures. Electrochemistry-grade *n*-Bu_4_NBF_4_ electrolyte was purchased from Sigma Aldrich. The glassy carbon electrodes were cleaned by first cycling at positive potentials in 1 m hydrochloric acid using a silver wire pseudo reference electrode and then polishing using alumina powder (1 μm diameter).

### Physical measurements

NMR spectra were recorded on a Bruker DPX-400 MHz spectrometer and the spectra were referenced against the solvent peak. The mass spectrum of ‘S_2_Se_2_’_pre_ was recorded by the University of Cambridge Mass Spectrometry Service using a Bruker Bio Apex 4.0 FTICR ESI-MS. The mass spectra of the metal complexes were recorded on a Waters Quattro LC electrospray ionization mass spectrometer. Expected and experimental isotope distributions of the compounds were compared. Elemental analysis was carried out by the microanalysis service of the Department of Chemistry, University of Cambridge. FTIR spectra were recorded on a Thermoscientific Nicolet iS50 FTIR spectrometer with an ATR sampling accessory. Electronic absorption spectra were recorded on an Agilent Cary UV-Vis 50 Bio spectrometer. The SEM images and EDX spectra were recorded using a Philips XL30 132–10 electron microscope. EDX studies (edax PV7760/68 ME) were run at a 15 kV acceleration voltage, spot size 4.0, and an acquisition time of at least 100 s. The elements were assigned and atomic ratios were identified using the built-in software (EDAX). XPS data were obtained at the National EPSRC XPS User’s Service (NEXUS) at Newcastle University, UK, an EPSRC Mid-Range Facility. Analysis was performed using a Kα spectrometer (Thermo Scientific, East Grinstead, UK) utilizing a monochromatic Al Kα X-ray source (1486.6 eV, 400 μm spot size, 36 W).

### X-ray crystallographic studies

Data were recorded with Mo Kα radiation (*λ*=0.71073 Å) on a Nonius Kappa CCD diffractometer fitted with an Oxford Cryosystems Cryostream cooling apparatus. The single crystal was mounted in Paratone N oil on the tip of a glass fiber and kept under a stream of N_2_. Structure solution was carried out using direct methods and refined by least squares (SHELXL-97)[27] using Chebyshev weights on *F*_o_^2^. The weighted *R*-factor *wR* and goodness of fit (GOF) are based on *F* ^2^. Crystal data, data collection parameters, and structure refinement details for the complexes are given in the Supporting Information, Table S1. The structure of complex [Ni(‘S_2_Se_2_’)] contained two crystallographically independent molecules in the asymmetric unit. A poorly resolved pentane solvent molecule co-crystallized with [NiFe(‘S_2_Se_2_’)(CO)_3_] and it was modeled as one half-weight molecule disordered about an inversion center with geometric restraints and a common isotropic displacement parameter for the carbon atoms. Selected bond distances and angles are shown in Tables 1 and 2. The mean bond distances and angles for the discussion in the paper were calculated as follows: for a sample of *n* observations *x_i_*, a weighted mean value (*x*_u_) with its standard deviation (σ) was calculated using the following equations: *x*_u_=Σ_*i*_*x_i_*/*n*, σ={Σ_*i*_(*x_i_*−*x*_u_)^2^/[*n*(*n*−1)]}^1/2^. Crystal structure images were created using Ortep 3 for Windows.[28] CCDC 1050563 http://www.ccdc.cam.ac.uk/cgi-bin/catreq.cgi([Ni(‘S_2_Se_2_’)]) and CCDC 1050564 http://www.ccdc.cam.ac.uk/cgi-bin/catreq.cgi([NiFe(‘S_2_Se_2_’)(CO)_3_]⋅0.5 C_5_H_12_) contain the supplementary crystallographic data for this paper. These data can be obtained free of charge from The Cambridge Crystallographic Data Centre via http://www.ccdc.cam.ac.uk/data_request/cif.

### Electrochemical measurements

Voltammograms were recorded at room temperature under inert gas using an IviumStat or CompactStat potentiostat. A standard three-electrode cell was used for all measurements with a glassy carbon disk working (3 mm diameter), a platinum mesh counter, and a Ag/Ag^+^ (organic solutions) or Ag/AgCl/KCl_(sat)_ (aqueous solutions) reference electrode. For voltammograms recorded in organic solvents containing *n*-Bu_4_NBF_4_ (0.1 m), the Fc^+^/Fc couple was used as a reference. For voltammograms recorded in a pH 7 aqueous phosphate solution (0.1 m), potentials were converted to the normal hydrogen electrode (NHE) by adding 0.2 V to the potential against Ag/AgCl/KCl_(sat)_.[29] Unless otherwise stated, the second of consecutive scans is shown, as currents were diffusion limited on this scan and all subsequent scans were identical.

For deposition of the films for characterization, a glassy carbon slide (1 cm x 1 cm x 0.1 cm) was immersed in a solution of [NiFe(‘S_2_Se_2_’)(CO)_3_] or [NiFe(‘S_4_’)(CO)_3_] (1 mm) in the presence of TFA (10 mm) in DMF with *n*-Bu_4_NBF_4_ (0.1 m). An electrode surface area of 1.6 cm^2^ was in contact with the electrolyte solution. A potential of approximately −1.75 V vs. Fc^+^/Fc was applied for 0.5 h. The modified electrode was then removed from the solution and rinsed with DMF (3 mL).

Catalytic films for controlled potential electrolysis were deposited from a solution of [NiFe(‘S_2_Se_2_’)(CO)_3_] or [NiFe(‘S_4_’)(CO)_3_] (1 mm) in the presence of TFA (10 mm) in DMF with *n*-Bu_4_NBF_4_ (0.1 m) on a glassy carbon or FTO-coated glass electrode (geometric surface area in contact with electrolyte solution of approximately 1.6 cm^2^) at −1.75 V vs. Fc^+^/Fc for 0.5 h. The modified electrode was then removed from the solution, rinsed with DMF (3 mL) and immersed into an aqueous phosphate solution (0.1 m, pH 7). Controlled potential electrolysis was carried out in an airtight electrochemical cell containing N_2_ with 2 % methane as internal standard for gas chromatography (GC) analysis. The headspace gas was analyzed using an Agilent 7890 A GC equipped with a 5 Å molecular sieve column, using N_2_ carrier gas with a flow rate of approximately 3 mL min^−1^. The GC columns were kept at 40 °C and a thermal conductivity detector was used.

### Synthesis and characterization

***Synthesis of ‘S***_***2***_***Se***_***2***_***’***_***pre***_: A solution of selenourea (1.40 g, 11.4 mmol) in ethanol (25 mL) was added to a solution of bis(3-chloro-2,2-methyl-1-thiapropyl)-*o*-xylene (2.00 g, 5.7 mmol) in ethanol (10 mL) and the colorless solution was refluxed for 30 min, during which time a white solid precipitated. The reaction mixture was cooled on ice, the solid product was isolated by filtration, washed with cold ethanol (3×5 mL) and diethyl ether (3×5 mL), and dried under high vacuum at room temperature. Yield: 1.91 g, 61 %. ^1^H NMR (400 MHz, D_2_O): *δ*=7.45 (2 H, m, Ar), 7.35 (2 H, m, Ar), 4.07 (4 H, s, CH_2_), 3.62 (4 H, s, CH_2_), 1.57 (12 H, s, CH_3_); ^13^C NMR (400 MHz, D_2_O): *δ*=168.15 (*C*(NH)NH_2_), 135.34 (Ar), 131.00 (Ar), 128.37 (Ar), 46.54 (*C*Me_2_), 41.31 (CH_2_), 30.17 (CH_2_), 27.61 ppm (Me); ATR-IR: 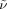
=3011, 2960, 1630, 1416, 693 cm^−1^; ESI-MS (H_2_O) +ve: 527 (100 %, C_18_H_31_N_4_S_2_Se_2_^+^); elemental analysis calcd (%) for C_18_H_32_Cl_2_N_4_S_2_Se_2_: C 36.19, H 5.40, Cl 11.87, N 9.38; found: C 36.28, H 5.30, Cl 11.97, N 9.08.

***Synthesis of [Ni(‘S***_***2***_***Se***_***2***_***’)]***: A solution of NMe_4_OH⋅5 H_2_O (121 mg, 668 μmol) in ethanol (4 mL) was added to a suspension of ‘S_2_Se_2_’_pre_ (200 mg, 335 μmol) in ethanol (4 mL) and the reaction mixture was stirred for 10 min until the white solid had dissolved. The solution was then added to a suspension of [Ni(acac)_2_] (86 mg, 335 μmol) in ethanol (20 mL), and the reaction mixture was heated to reflux for 1 h, during which time a green solution had formed. The solvent volume was reduced to 10 mL and the resulting green precipitate was separated by filtration, washed with ethanol (3×3 mL), and dried under vacuum. The product was recrystallized by slow liquid diffusion of hexane into a dichloromethane solution of the complex. Yield: 138 mg, 83 %. ^1^H NMR (400 MHz, CD_2_Cl_2_): *δ*=7.33 (2 H, m, Ar), 7.28 (2 H, m, Ar), 3.90 (4 H, s, CH_2_), 2.36 (4 H, s, CH_2_), 1.73 ppm (12 H, s, CH_3_); ^13^C NMR (400 MHz, CD_2_Cl_2_): *δ*=134.43 (Ar), 131.32 (Ar), 129.70 (Ar), 65.67, 33.68, 28.34, 26.90 ppm; ESI-MS (CHCl_3_) +ve: 498 (100 %, [*M*]^+^); elemental analysis calcd (%) for C_16_H_24_NiS_2_Se_2_: C 38.66, H 4.87; found: C 38.67, H 4.68; *λ*_max_ (CH_2_Cl_2_)=290 nm (*ε*=17.2×10^3^ L mol^−1^ cm^−1^). Single crystals for X-ray analysis were grown from liquid diffusion of hexane into a dichloromethane solution of the complex.

***Synthesis of [NiFe(‘S***_***2***_***Se***_***2***_***’)(CO)***_***3***_***]***: A solution of [Fe(CO)_3_(bda)] (50 mg, 174 μmol) in dichloromethane (3 mL) was added to a solution of [Ni(‘S_2_Se_2_’)] (86 mg, 174 μmol) in dichloromethane (3 mL) and the resulting red solution was stirred at room temperature. The reaction was followed by monitoring the disappearance of the carbonyl-stretches in [Fe(CO)_3_(bda)] at 2069, 2007, and 1988 cm^−1^ by ATR-IR spectroscopy. The reaction was complete after approximately 1 h, whereupon the solvent was removed under high vacuum and the product was purified using column chromatography (SiO_2_, dichloromethane) to give a red solid. Yield: 48 mg, 43 %. ^1^H NMR (400 MHz, CD_2_Cl_2_): *δ*=7.52 (1 H, m, Ar), 7.36 (3 H, m, Ar), 4.84 (1 H, d, CH_2_), 4.24 (1 H, d, CH_2_), 3.97 (1 H, d, CH_2_), 3.82 (1 H, d, CH_2_), 3.10 (1 H, d, CH_2_), 3.01 (1 H, d, CH_2_), 2.80 (1 H, d, CH_2_), 2.72 (1 H, d, CH_2_), 1.87 (3 H, s, CH_3_), 1.85 (3 H, s, CH_3_), 1.63 (3 H, s, CH_3_), 1.41 ppm (3 H, s, CH_3_); ^13^C NMR (400 MHz, CD_2_Cl_2_): *δ*=219.12 (CO), 134.33 (Ar), 133.68 (Ar), 132.65 (Ar), 131.40 (Ar), 129.19 (Ar), 129.06 (Ar), 66.16 (*C*Me_2_), 61.71 (*C*Me_2_), 39.98 (CH_2_), 35.88 (CH_2_), 34.66 (CH_2_), 29.18 (CH_2_), 27.98 (Me), 27.57 (Me), 25.95 (Me), 25.52 ppm (Me); ATR-IR: 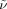
=2000 (CO), 1942 (CO), 1835 cm^−1^ (CO); ESI-MS (CH_2_Cl_2_) +ve: 638 (100 % [*M*]^+^); elemental analysis calcd (%) for C_19_H_24_FeNiO_3_S_2_Se_2_: C 35.83, H 3.80; found: C 36.53, H 3.81; *λ*_max_ (DMF)=399 nm (*ε*=5.79×10^3^ L mol^−1^ cm^−1^). Single crystals were grown from liquid diffusion of pentane into a dichloromethane solution of the complex.

## Supporting Information

As a service to our authors and readers, this journal provides supporting information supplied by the authors. Such materials are peer reviewed and may be re-organized for online delivery, but are not copy-edited or typeset. Technical support issues arising from supporting information (other than missing files) should be addressed to the authors.

miscellaneous_information
