# Supplementary material for: Synthetic Active Site Model of the [NiFeSe] Hydrogenase
Source: Chemistry. 2015 Apr 2;21(22):8096–104. doi: 10.1002/chem.201500311 (PMC4510704; doi:10.1002/chem.201500311)
Supplement: Supplementary file 1 — miscellaneous_information [file chem0021-8096-sd1.pdf]

CHEMISTRY
A European Journal

Supporting Information

Synthetic Active Site Model of the [NiFeSe] Hydrogenase

Claire Wombwell and Erwin Reisner*[a]

chem_201500311_sm_miscellaneous_information.pdf

Supporting Information

Synthetic Active Site Model of the [NiFeSe] Hydrogenase

Claire Wombwell and Erwin Reisner*

Christian Doppler Laboratory for Sustainable SynGas Chemistry, Department of Chemistry, University of Cambridge, Lensfield Road, Cambridge CB2 1EW, U.K.

*Corresponding author: reisner@ch.cam.ac.uk

Table S1. Crystal data and structure refinement details for [Ni('S_2Se_2')] and [NiFe('S_2Se_2')$(CO)_3$].

	[Ni('S_2Se_2')]	[NiFe('S_2Se_2')$(CO)_3$]· ½C_5H_{12}
Empirical formula	$C_{16}H_{24}NiS_2Se_2$	$C_{21.5}H_{30}FeNiO_3S_2Se_2$
Formula weight	497.10	673.06
Temperature (K)	180(2)	180(2)
Space group	$P2_1/n$	$P2_1/n$
a (Å)	13.3707(6)	10.5460(2)
b (Å)	17.4020(9)	17.6154(4)
c (Å)	16.3398(8)	13.7094(3)
β (deg)	90.471(2)	93.898(1)
V (Å^3)	3801.8(3)	2540.93(9)
Z	8	4
φ_{calc} (g cm^{-3})	1.737	1.759
μ (Mo Kα, mm^{-1})	7.818	4.362
Crystal size (mm^3)	0.18 x 0.17 x 0.16	0.28 x 0.28 x 0.21
Θ range, deg	8.11 to 27.06	3.63 to 32.01
Total number of data	34027	24514
Number of unique data	4588	8452
Number of parameters	387	274
Completeness	0.995	0.956
$R1^a$	0.0311	0.0298
$wR2^b$	0.0744	0.0583

$R1 = \sum||F_o|-|F_c||/\sum|F_o|$ (b) $wR2 = \{\sum [w(F_o^2-F_c^2)^2]/ \sum [w(F_o^2)^2]\}^{1/2}$

Table S2. Electronic absorption data for [NiFe('S_2Se_2')$(CO)_3$] and [NiFe('S_4')$(CO)_3$] in DMF (sh = shoulder).

[NiFe('S_2Se_2')$(CO)_3$]		[NiFe('S_4')$(CO)_3$]	
λ_{max} / nm	ε / M^{-1} cm^{-1}	λ_{max}/ nm	ε / M^{-1} cm^{-1}
341 (sh)	5797	330	8043
399	3303	393	5974
442 (sh)	2964	432 (sh)	5372
608 (sh)	782	502 (sh)	2981
703	260	698	390

Table S3. EDX and XPS data of heterogeneous catalyst films formed upon electrodeposition of [NiFe('S_2Se_2')$(CO)_3$] and [NiFe('S_4')$(CO)_3$] onto a glassy carbon slide. To form the deposit, the slide (1.6 cm^2 surface area in contact with solution) was immersed in a solution of [NiFe('S_2Se_2')$(CO)_3$] or [NiFe('S_4')$(CO)_3$] (1 mM) in the presence of TFA (10 mM) in DMF with *n*–Bu_4NBF_4 (0.1 M). A redox potential of approximately –1.75 V *vs.* Fc^+/Fc was applied for 0.5 h (platinum mesh counter, Ag^+/Ag reference electrode). The modified electrode was then removed from the solution, rinsed with DMF (3 mL) and dried.

Element	[NiFe('S_2Se_2')$(CO)_3$] deposit (relative atomic %)		[NiFe('S_4')$(CO)_3$] deposit (relative atomic %)	
	EDX	XPS	EDX	XPS
Nickel	42	55	15	16
Iron	38	45	72	62
Sulfur	4	—[a]	13	22
Selenium	16	*n.d.*[b]	—	—

(a) Below level of quantification, (b) Not determinable as Se3p signal overlaps with Ni3p and Fe3p signals.

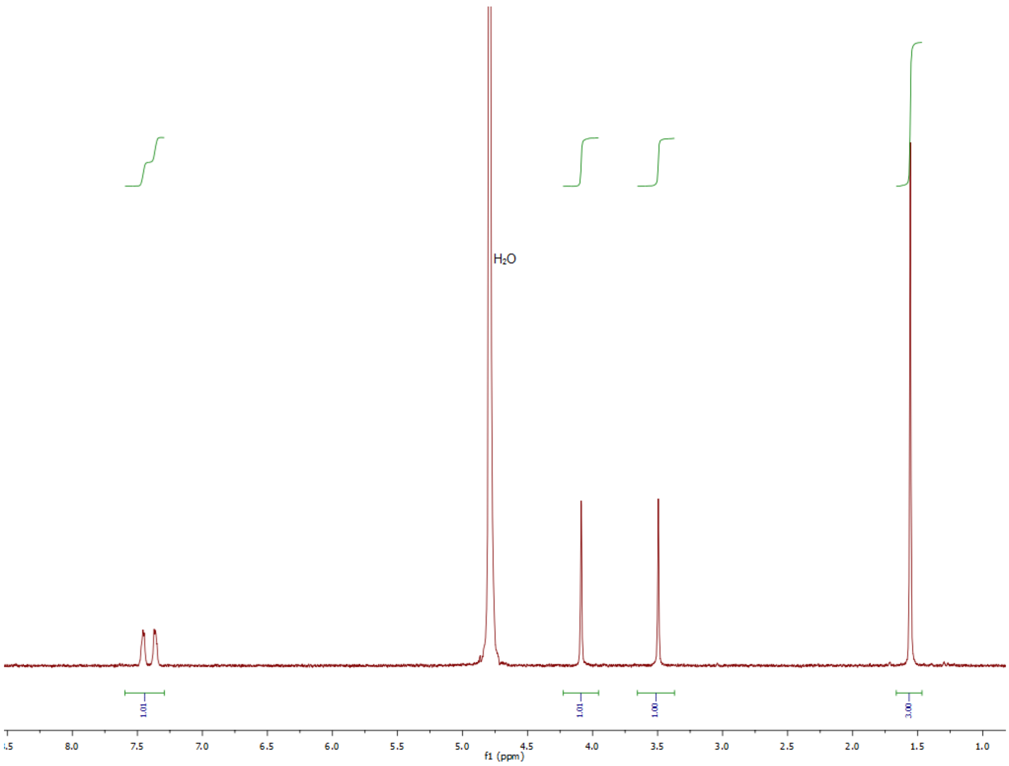

Figure S1. ^{1}H NMR spectrum of ‘S_2Se_2’$_{pre}$ in D_2O.

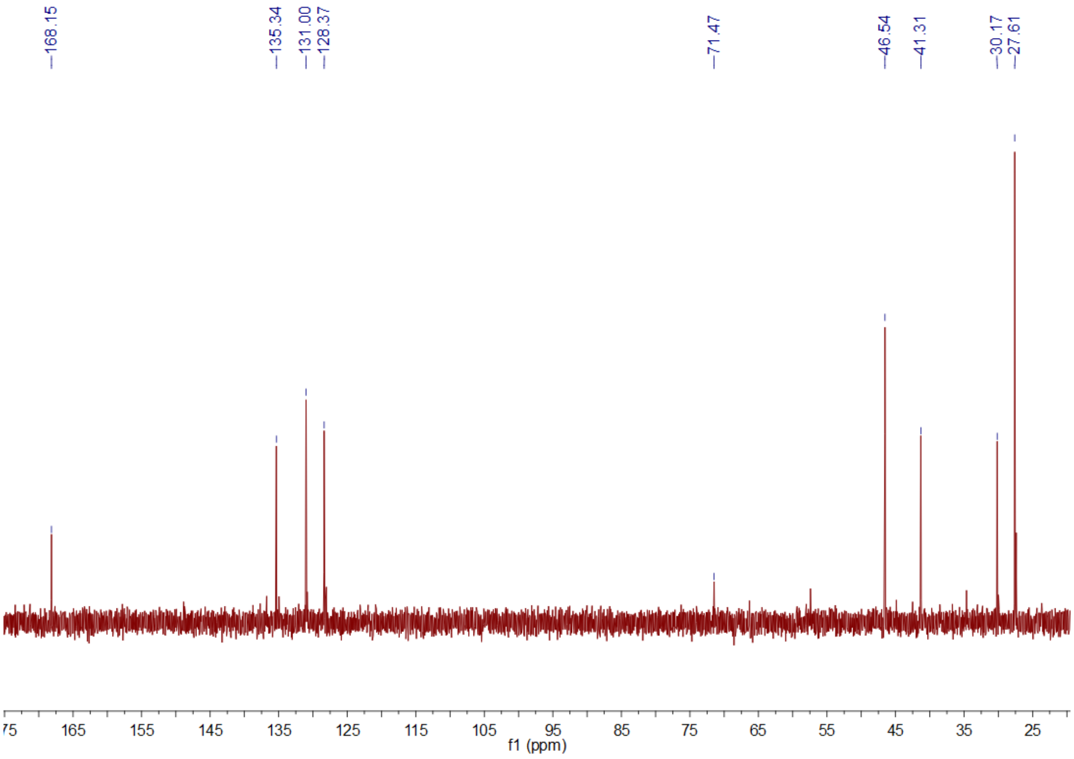

Figure S2. ^{13}C NMR spectrum of ‘S_2Se_2’$_{pre}$ in D_2O.

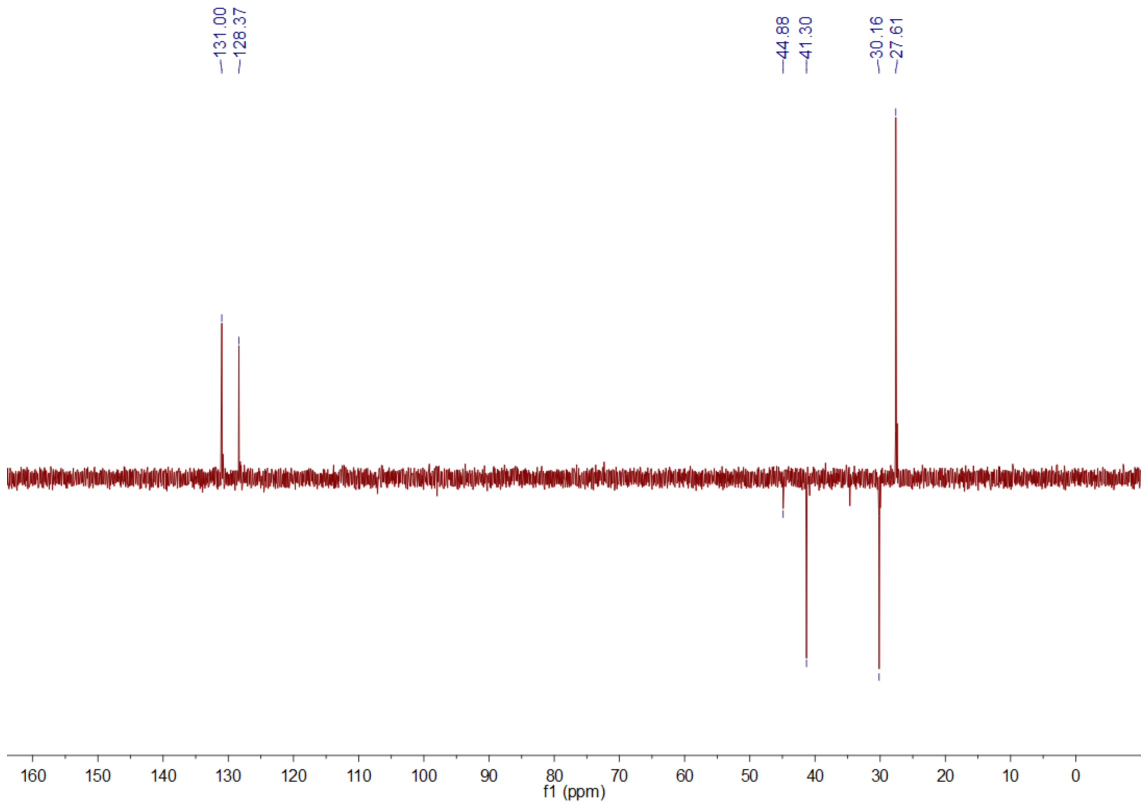

Figure S3. ^{13}C NMR DEPT spectrum of ‘S_2Se_2’$_{pre}$ in D_2O.

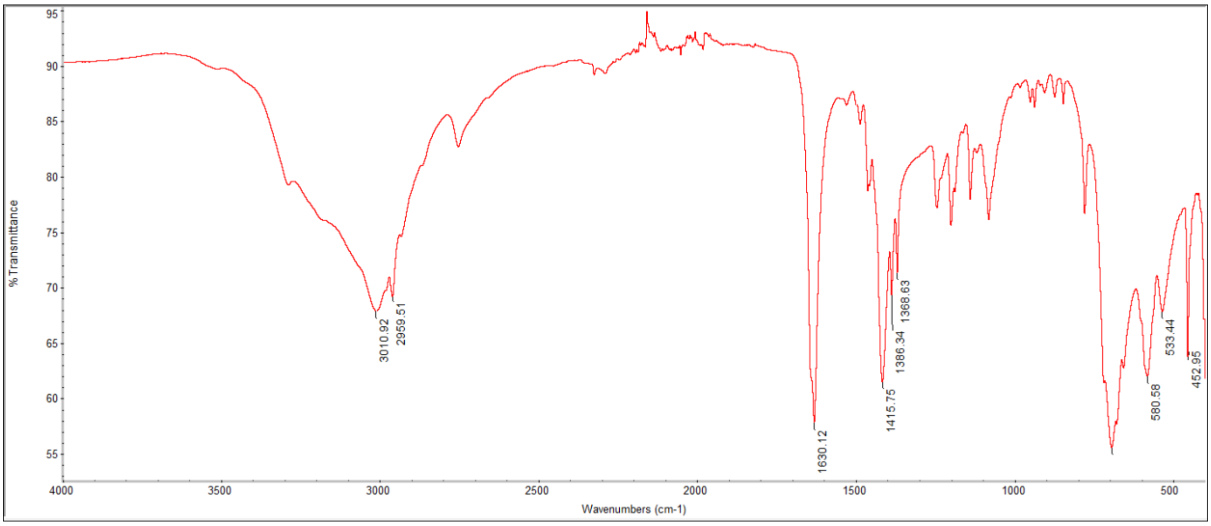

Figure S4. ATR–IR spectrum of ‘S_2Se_2’$_{pre}$.

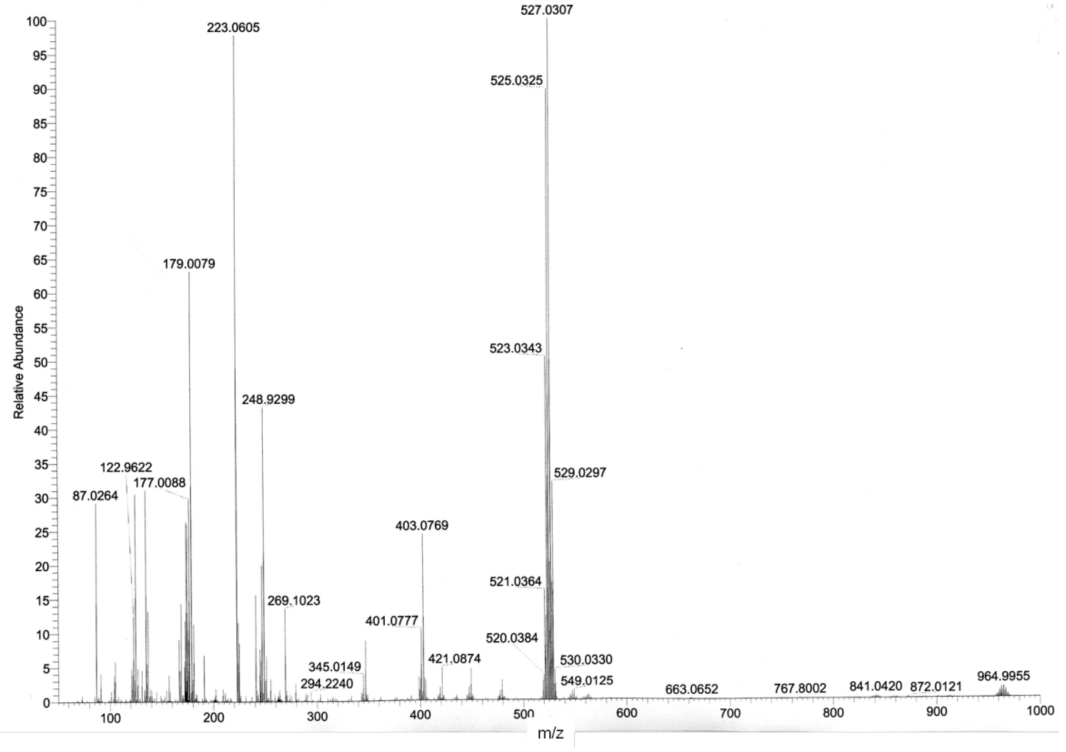

Figure S5. ESI–MS of ‘S_2Se_2’$_{pre}$ in H_2O (full spectrum).

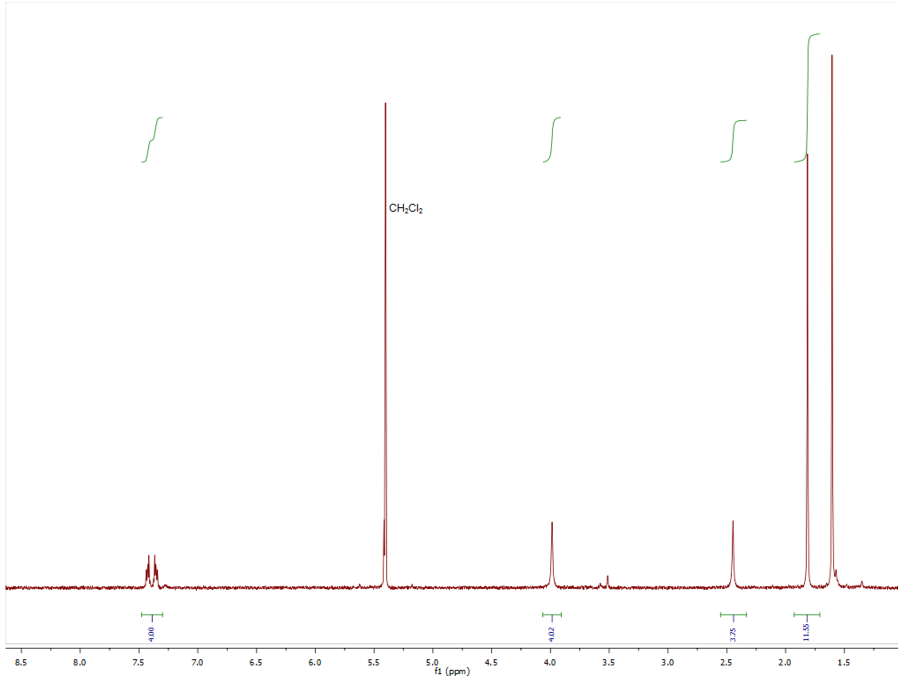

Figure S6. ^{1}H NMR spectrum of [Ni(‘S_2Se_2’)] in CD_2Cl_2.

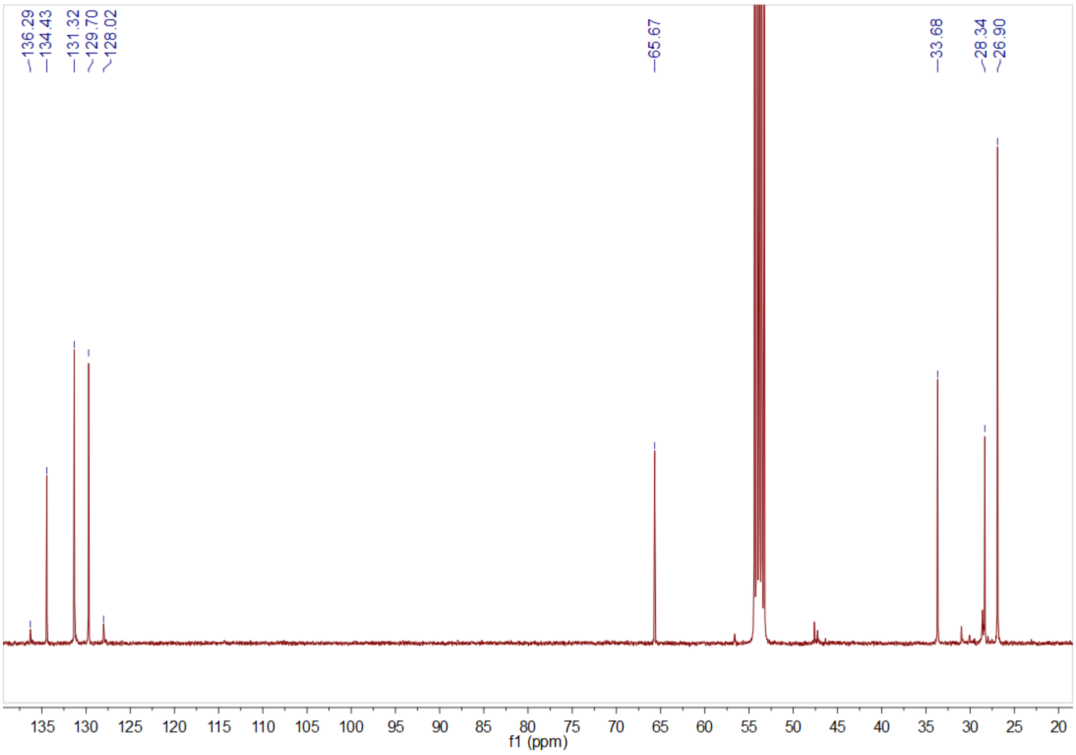

Figure S7. ^{13}C NMR spectrum of [Ni('S_2Se_2')] in CD_2Cl_2.

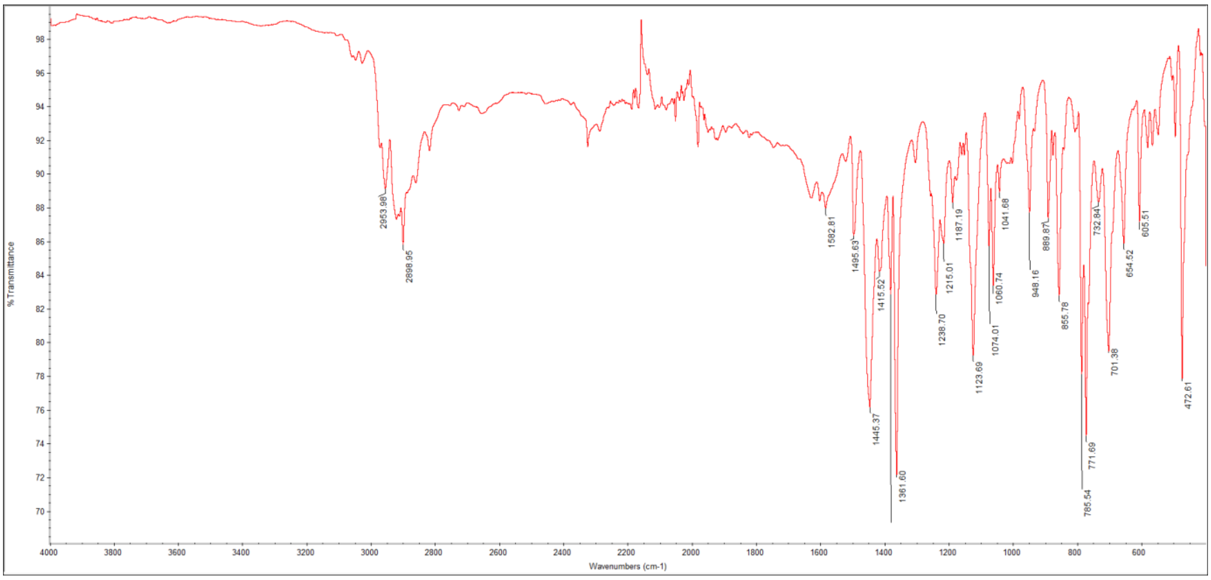

Figure S8. ATR–IR spectrum of [Ni('S_2Se_2')].

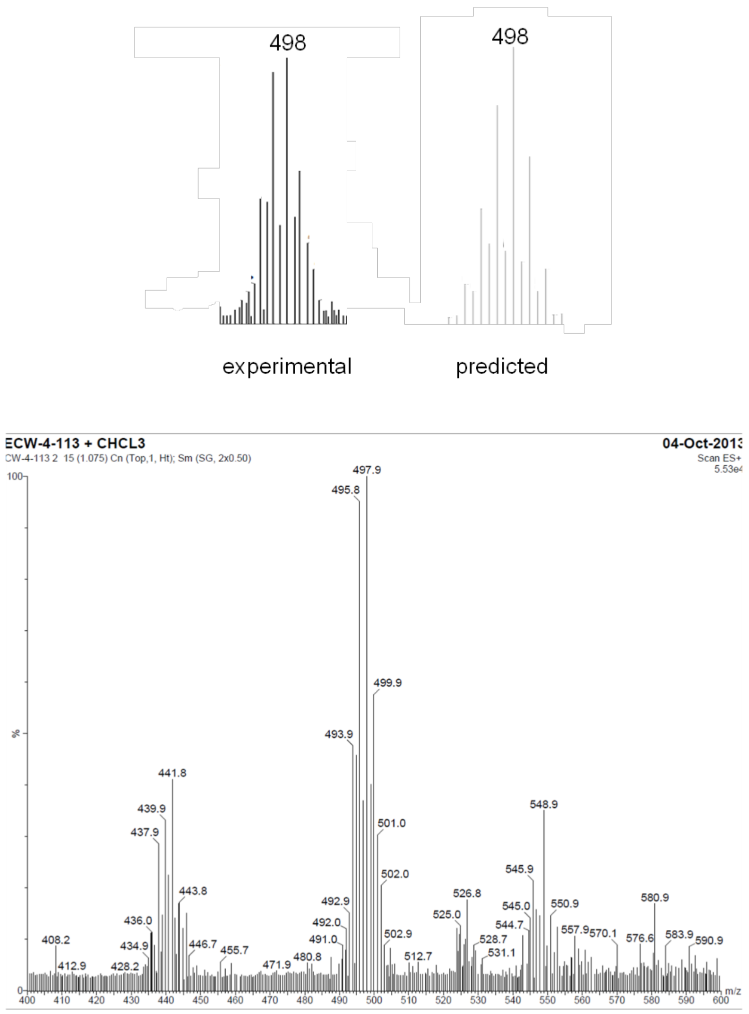

Figure S9. ESI–MS of [Ni('S_2Se_2')] in $CHCl_3$ (left) predicted spectrum (right) and full spectrum (bottom).

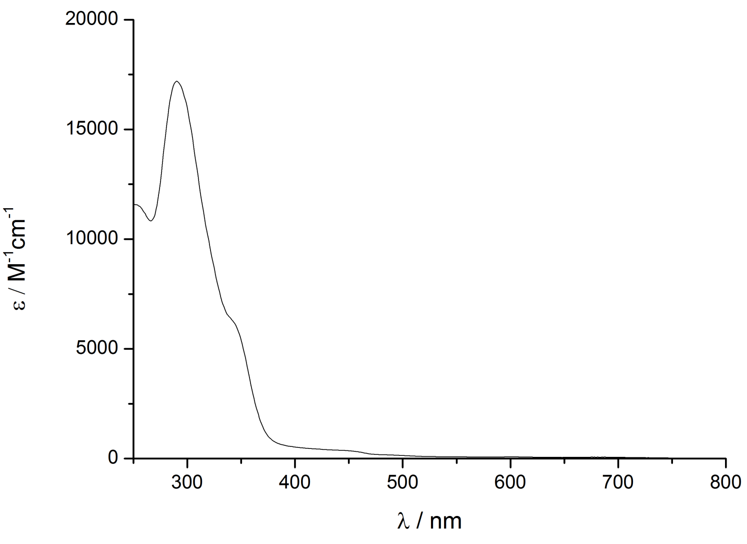

Figure S10. Electronic absorption spectrum of [Ni('S_2Se_2')] in CH_2Cl_2.

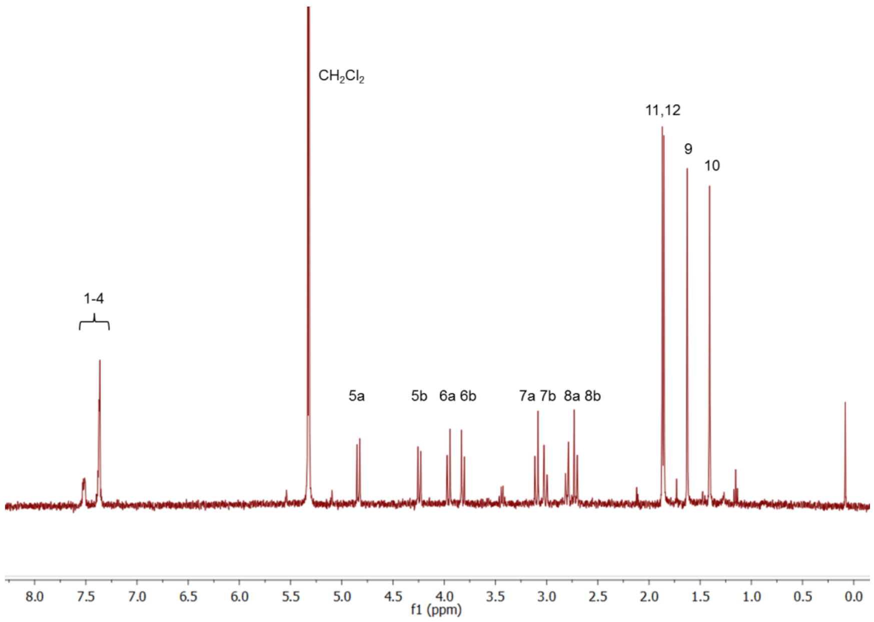

Figure S11. ^{1}H NMR spectrum of [NiFe('S_2Se_2')$(CO)_3$] in CD_2Cl_2 (aromatic protons labelled 1-4, methylene proton pairs labelled 5-8, methyl protons labelled 9-12).

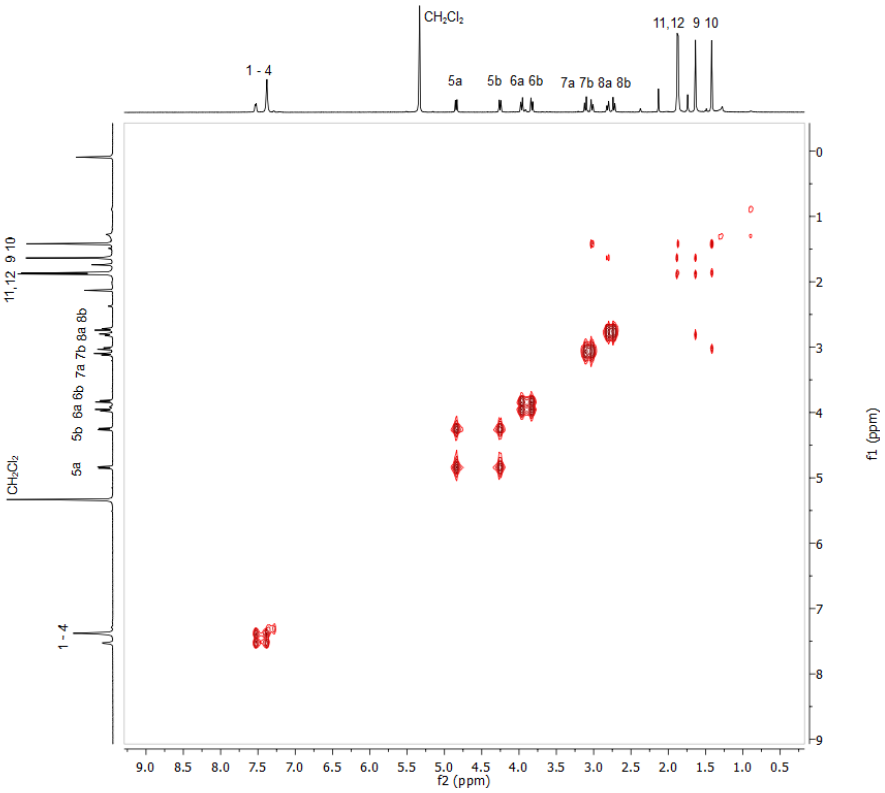

Figure S12. ^{1}H COSY NMR spectrum of [NiFe('S_2Se_2')$(CO)_3$] in CD_2Cl_2 (aromatic protons labelled 1-4, methylene proton pairs labelled 5-8, methyl protons labelled 9-12).

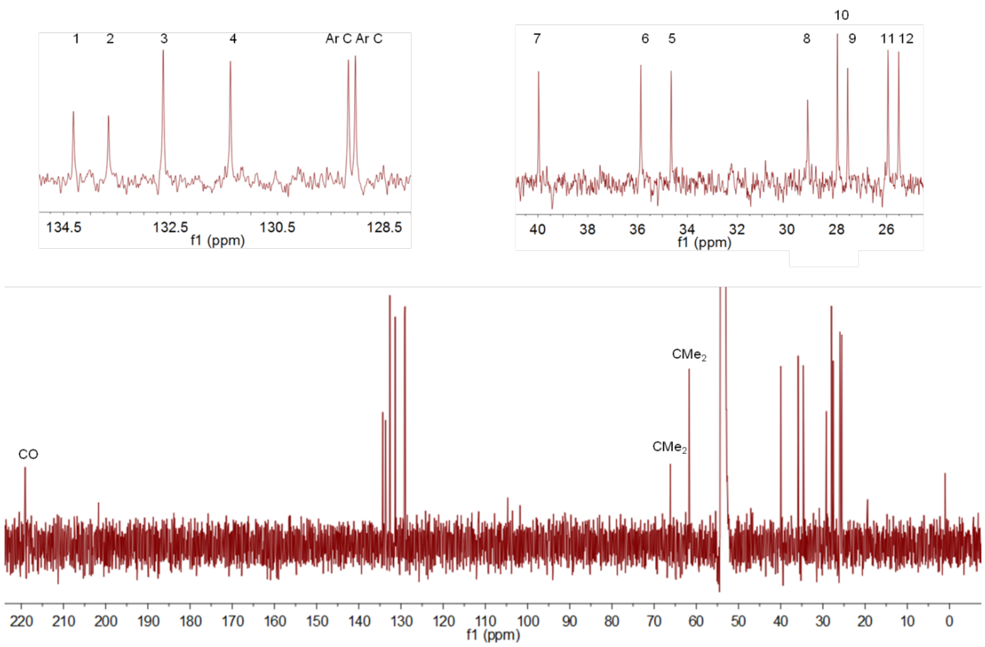

Figure S13. ^{13}C NMR spectrum of [NiFe('S_2Se_2')$(CO)_3$] in CD_2Cl_2 (numbering of signals assigns them to their corresponding proton signals).

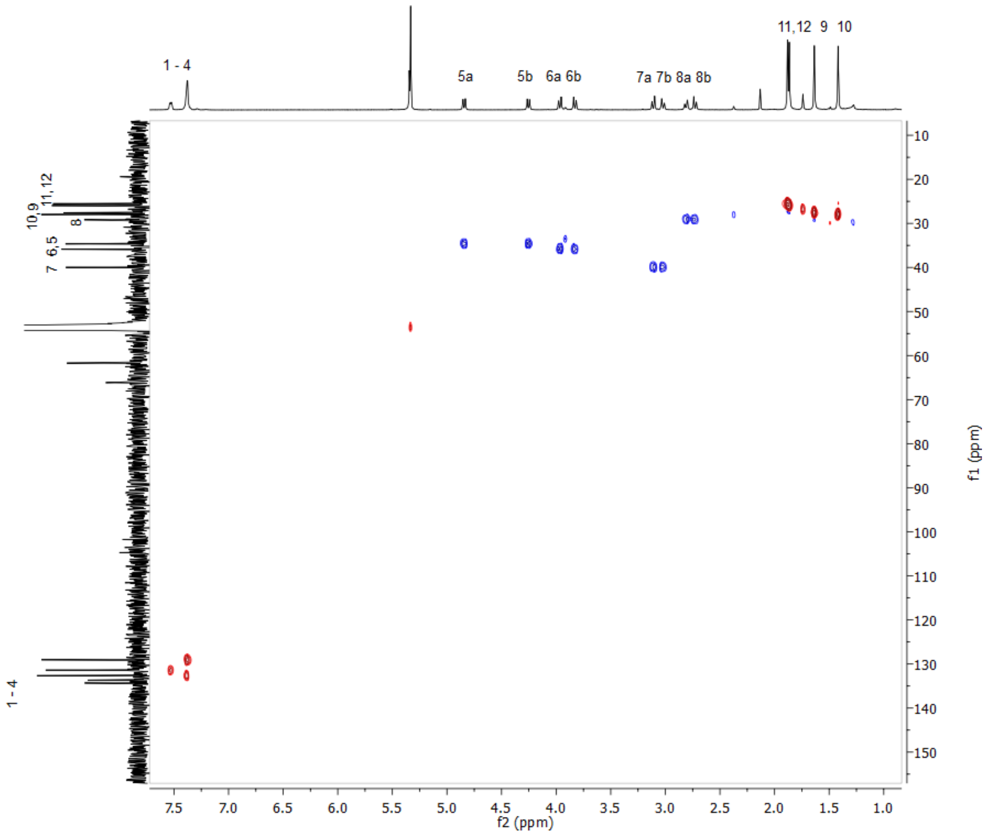

Figure S14. ^{1}H–^{13}C HMQC NMR spectrum of [NiFe('S_2Se_2')$(CO)_3$] in CD_2Cl_2.

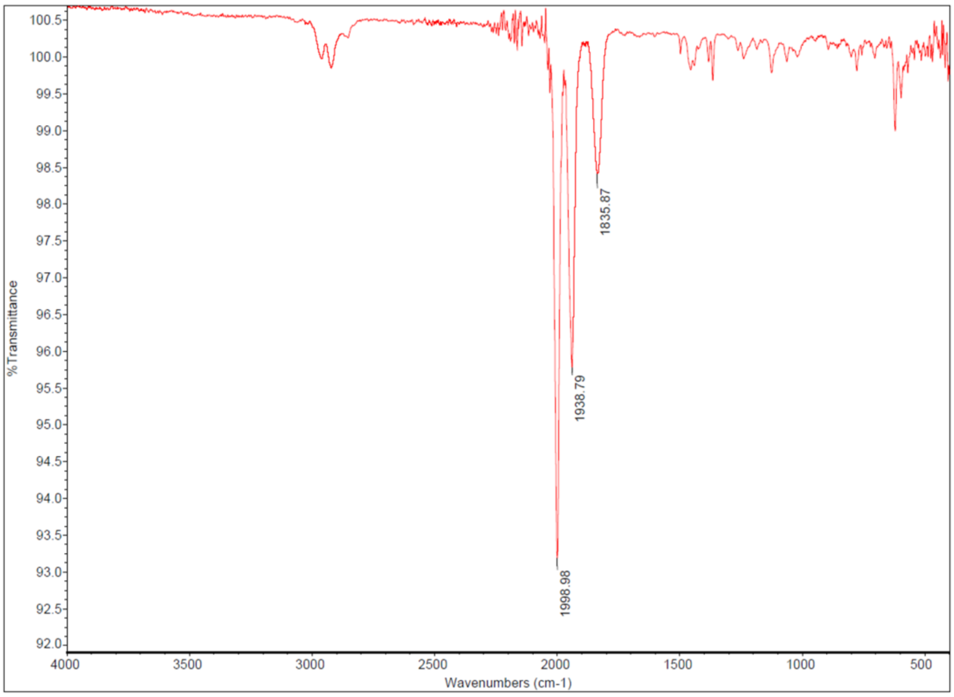

Figure S15. ATR–IR spectrum of [NiFe('S_2Se_2')$(CO)_3$].

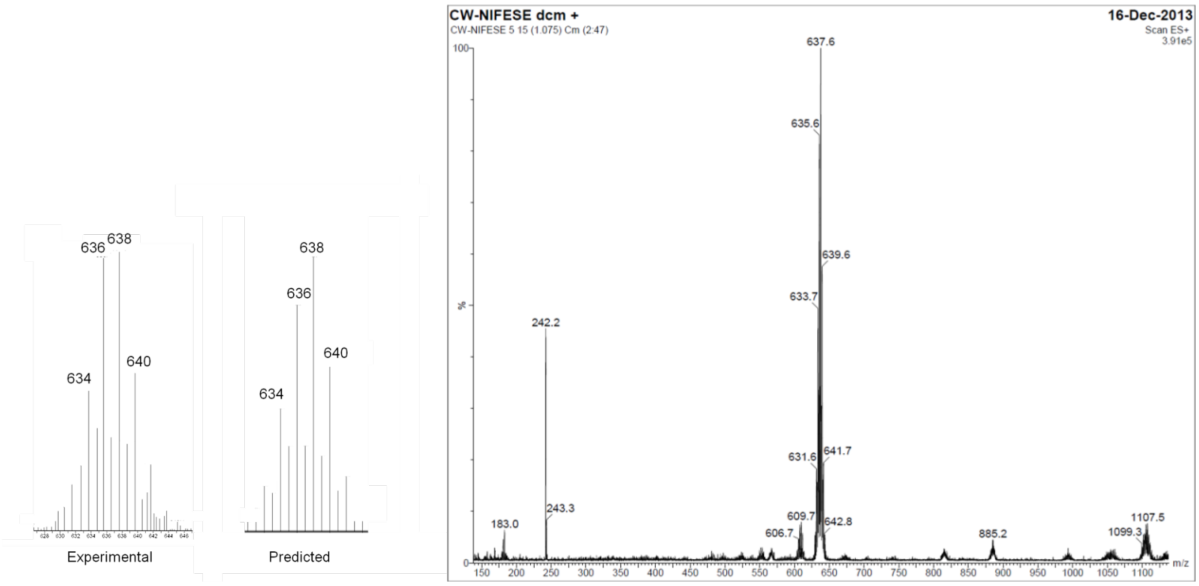

Figure S16. ESI–MS of [NiFe('S_2Se_2')$(CO)_3$] in dichloromethane (left), predicted spectrum of [NiFe('S_2Se_2')$(CO)_3$] (middle) and full spectrum (right).

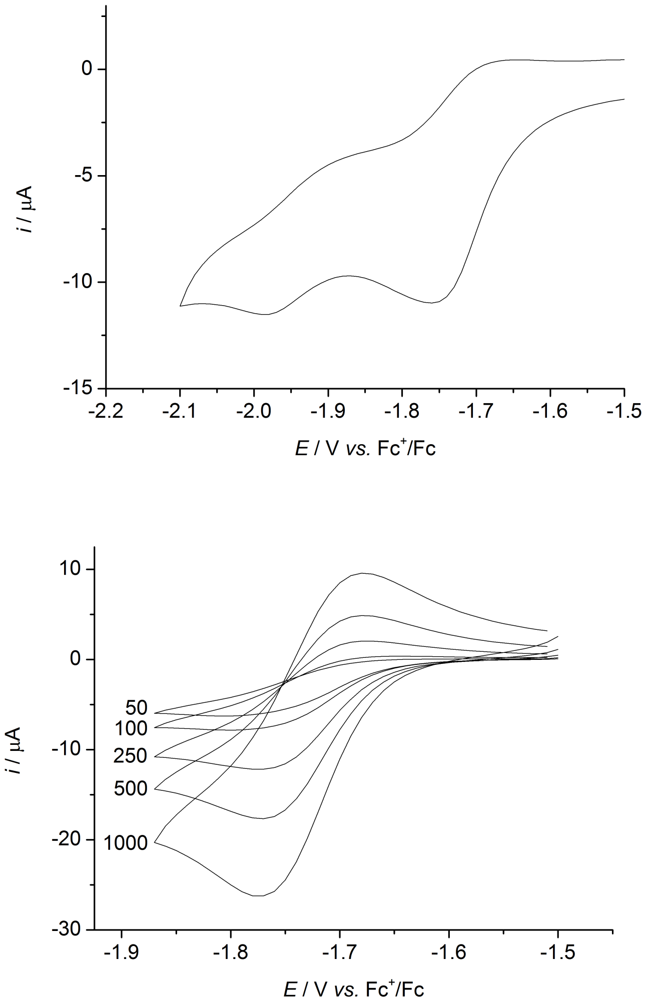

Figure S17. Cyclic voltammogram of [NiFe('S$_4$')(CO)$_3$] (1 mM) in acetonitrile with *n*–Bu$_4$NBF$_4$ (0.1 M). Top: scanning to negative potentials at 100 mV s^{-1} and bottom: only the first reduction wave at different scan rates (50–1000 mV s^{-1}).

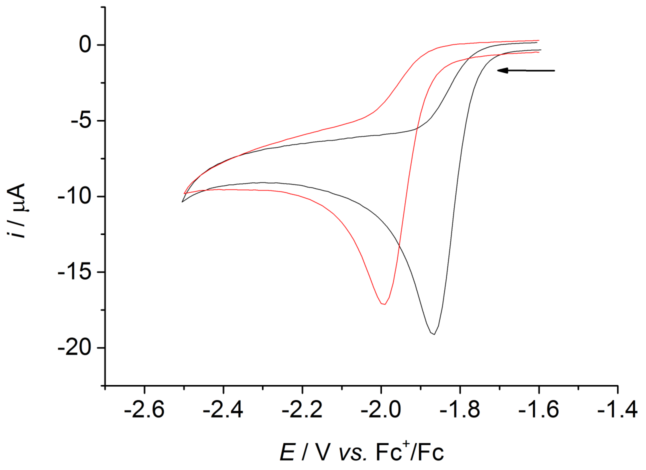

Figure S18. Cyclic voltammogram of [Ni('S_2Se_2')] (black trace) and [Ni('S_4')] (red trace) in DMF (1 mM) with *n*–Bu_4NBF_4 (0.1 M) at a scan rate of 100 mV s^{-1}.

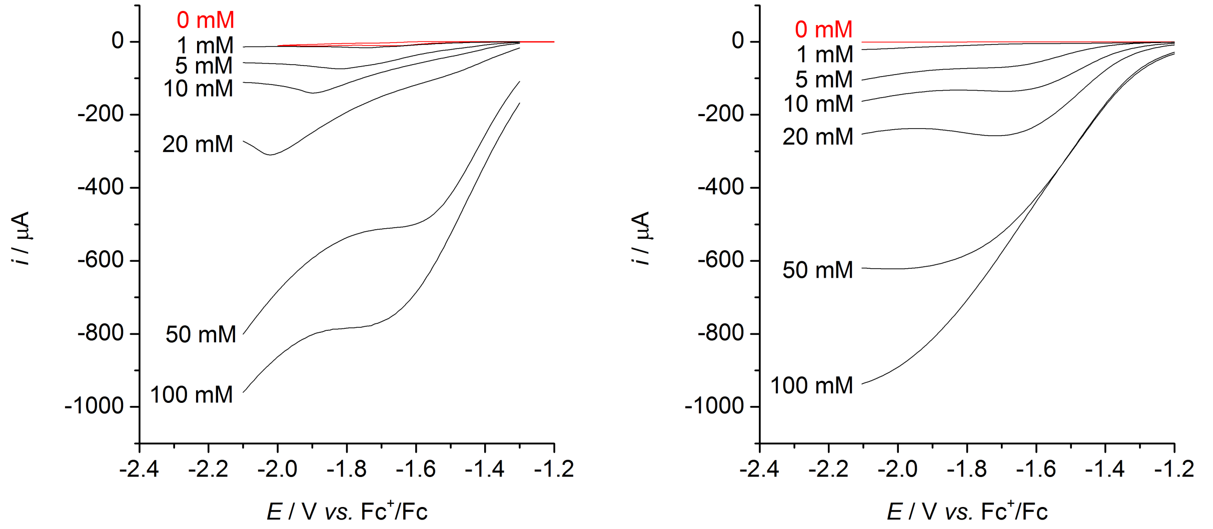

Figure S19. Linear sweep voltammograms of increasing concentrations of TFA in acetonitrile with *n*–Bu_4NBF_4 (0.1 M) on a glassy carbon working electrode in the presence (left) and in the absence (right) of [NiFe('S_4')$(CO)_3$] (1 mM).

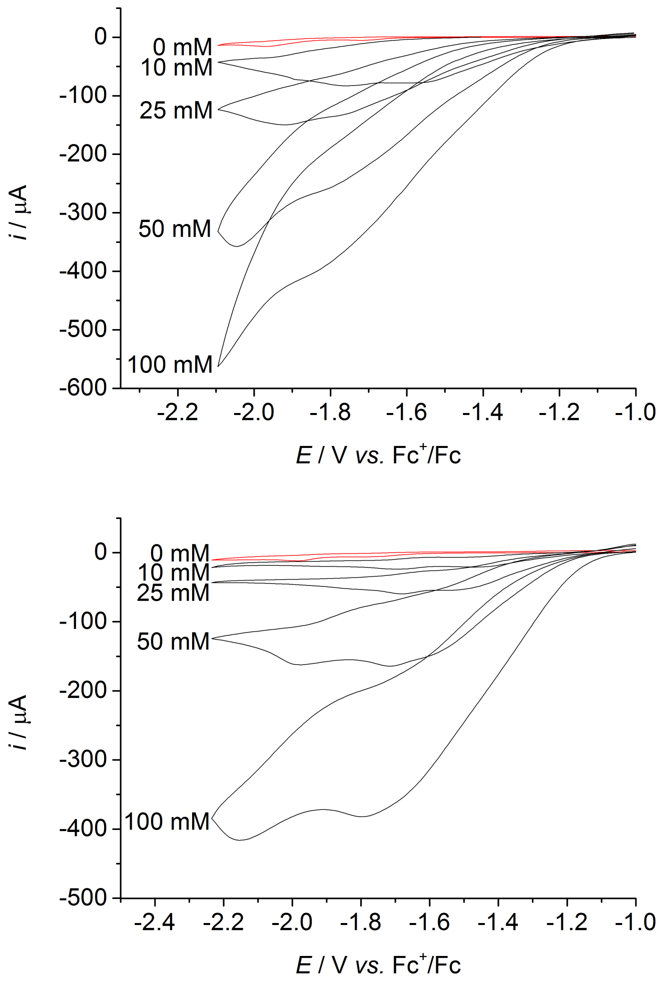

Figure S20. Cyclic voltammogram of [NiFe('S_2Se_2')$(CO)_3$] (1 mM, top) and [NiFe('S_4')$(CO)_3$] (1 mM, bottom) with increasing concentrations TFA in DMF (0.1 M *n*–Bu_4NBF_4).

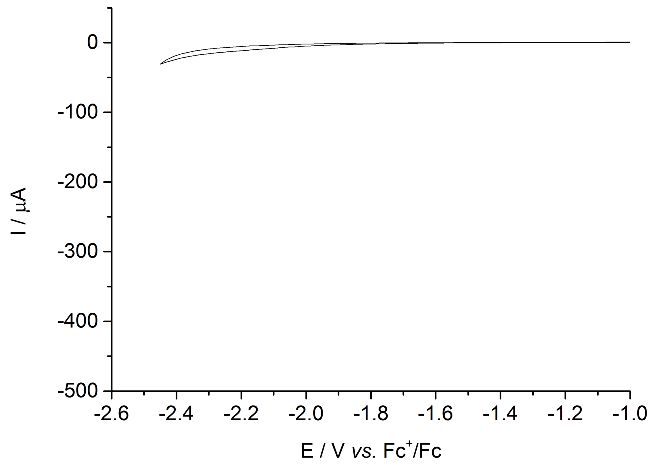

Figure S21. Cyclic voltammogram of 100 mM TFA in dimethyl formamide (0.1 M *n*–Bu_4NBF_4) on a glassy carbon electrode (no complex).

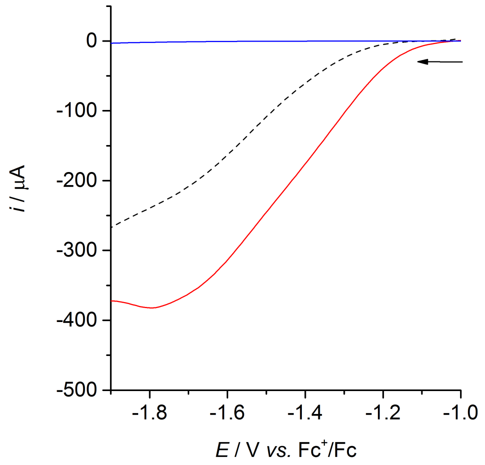

Figure S22. Linear sweep voltammogram of the solid deposit formed from electrodeposition of [NiFe('S_4')$(CO)_3$] in the presence (solid red trace) and in the absence (dashed black trace) of dissolved [NiFe('S_4')$(CO)_3$] (1 mM) on a glassy carbon working electrode and an unmodified glassy carbon working electrode (solid blue trace). The voltammograms were recorded in a fresh electrolyte solution containing TFA (100 mM) in DMF (0.1 M *n*–Bu_4NBF_4) at a scan rate of 100 mV s^{-1}.

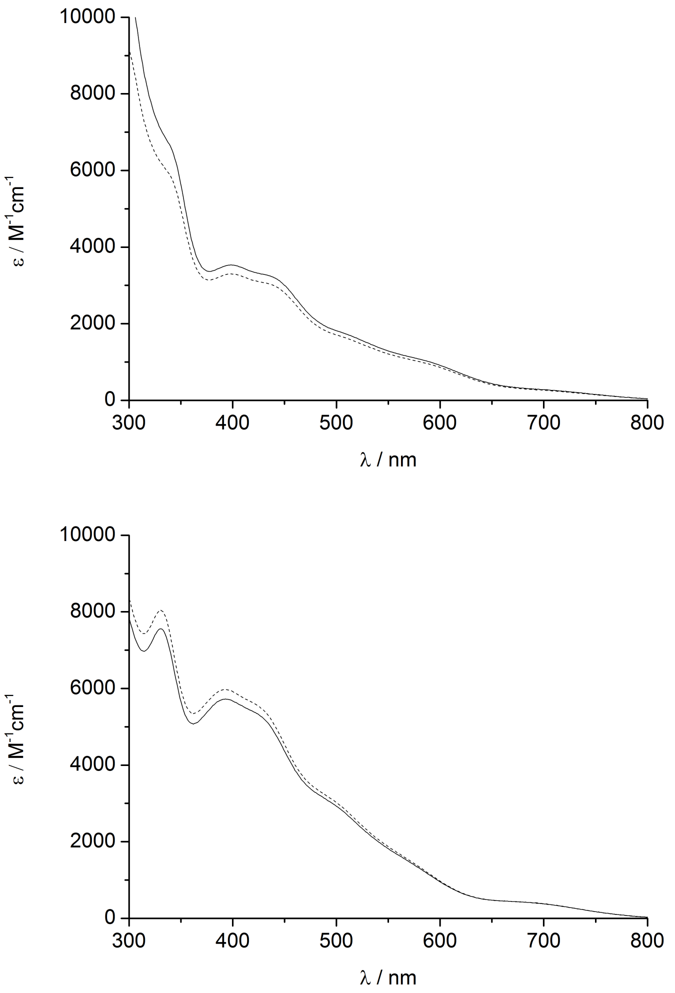

Figure S23. Electronic absorption spectra of [NiFe('S_2Se_2')$(CO)_3$] (top) and [NiFe('S_4')$(CO)_3$] (bottom) in DMF (0.1 mM) with (solid line) and without (dashed line) TFA (10 mM).

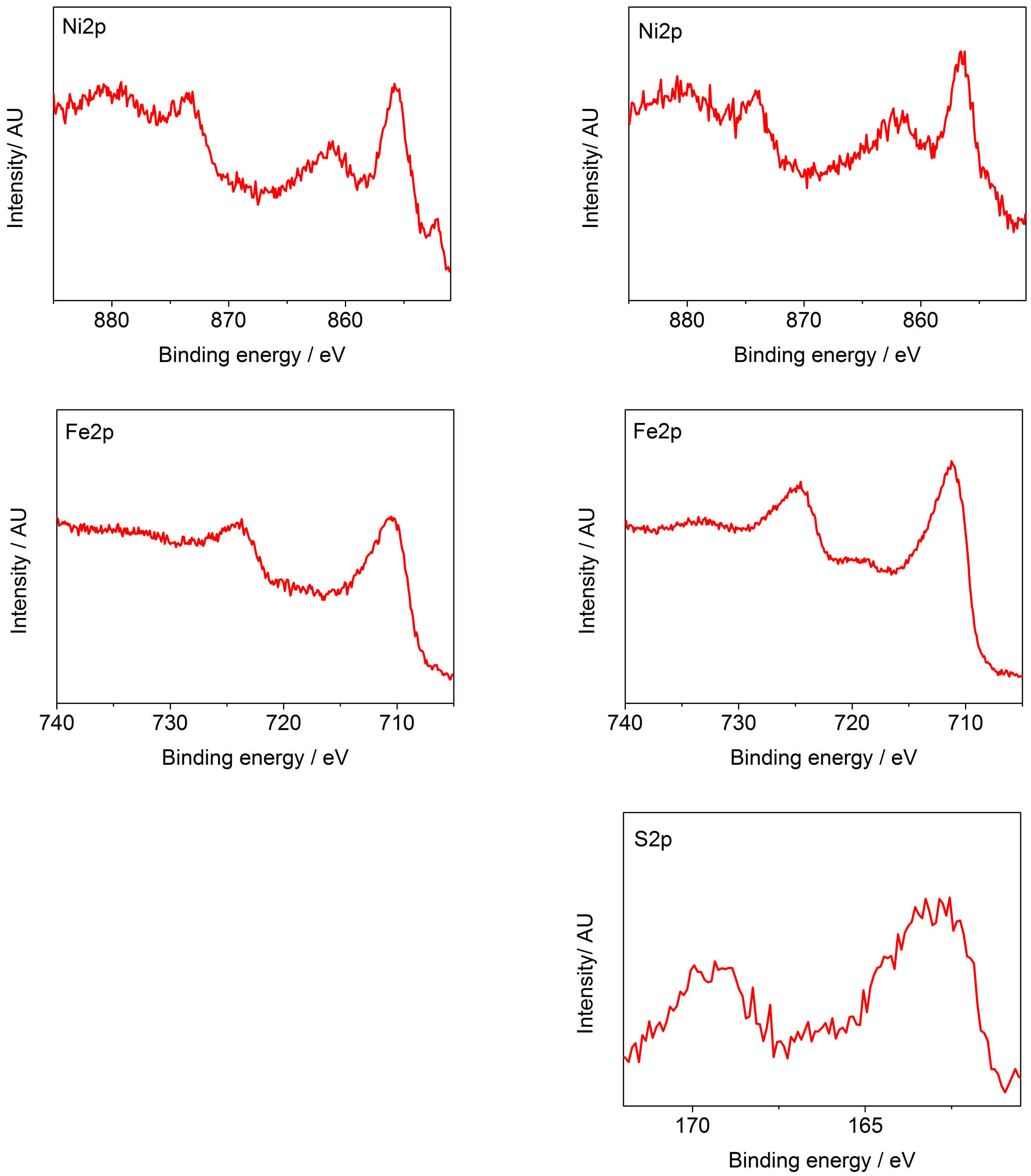

Figure S24. XPS analysis of films electrodeposited on a glassy carbon electrode from [NiFe('S_2Se_2')$(CO)_3$] (left) and [NiFe('S_4')$(CO)_3$] (right).

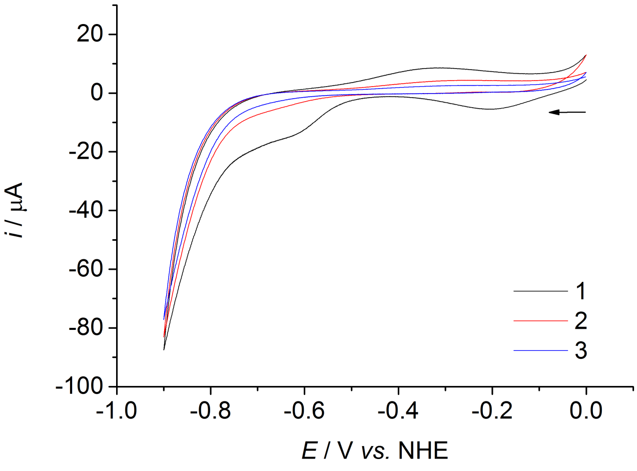

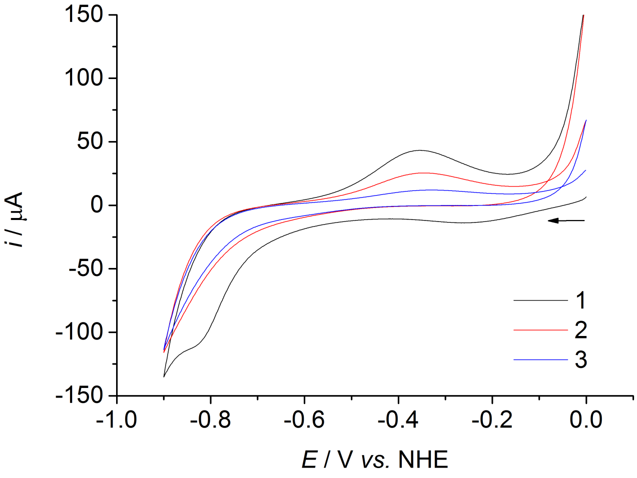

Figure S25. Cyclic voltammogram of deposits formed from [NiFe('S_2Se_2')$(CO)_3$] (top) and [NiFe('S_4')$(CO)_3$] (bottom) on a glassy carbon disc electrode (diameter 3 mm) in pH 7 phosphate buffered H_2O (0.1 M) at 100 mV s^{-1} showing the first three scans.

End of Supporting Information
